# *TP53* deficiency in AML induces resistance to T-cell engagers through an immunosuppressive secretome

**DOI:** 10.1038/s41375-026-02991-6

**Published:** 2026-06-01

**Authors:** Lis Winter, Lea Pawlowsky, Amelie Muth, Anne-Sophie Neumann, Kieron White, Maryam Kazerani, Daniel Nixdorf, Bettina Brauchle, Lisa Rohrbacher, Gerulf Hänel, Agnese Petrera, Eva Briem, Gordon V. Hoffmann, Thomas Janert, Emanuele Carlini, Adrian Gottschlich, Karsten Spiekermann, Tobias Straub, Roman Kischel, Sebastian Kobold, Michael Andreeff, Veit L. Buecklein, Marion Subklewe

**Affiliations:** 1 Laboratory for Translational Cancer Immunology, Gene Center, LMU Munich, Munich, Germany; 2 Department of Medicine III, LMU University Hospital, LMU Munich, Munich, Germany; 3 Metabolomics and Proteomics Core, Helmholtz Zentrum München, Germany; Research Center for Environmental Health (GmbH), Neuherberg, Germany; 4 Anthropology and Human Genomics, Faculty of Biology, LMU Munich, Munich, Germany; 5 Division of Clinical Pharmacology, LMU University Hospital, LMU Munich, Munich, Germany; 6 Bioinformatics Unit, Biomedical Center, LMU Munich, Martinsried, Germany; 7 Amgen Research (Munich) GmbH, Munich, Germany; 8 German Cancer Consortium (DKTK) Partner Site Munich, Munich, Germany; 9 German Center for Lung Research (DZL), Partner Site Munich, Munich, Germany; 10 Department of Leukemia, The University of Texas MD Anderson Cancer Center, Houston, TX USA; 11 German Cancer Consortium (DKTK), Partner Site Munich and German Cancer Research Center (DKFZ), Heidelberg, Germany; 12 Bavarian Cancer Research Center (BZKF), Munich, Germany

**Keywords:** Tumour immunology, Tumour immunology, Cancer immunotherapy, Cancer therapeutic resistance, Targeted therapies

## Abstract

Bispecific T-cell engagers (BiTE^®^ molecules) have transformed the treatment of B-cell malignancies, yet clinical activity in AML has been modest. Resistance is driven in part by the genetic heterogeneity of AML, most notably *TP53* mutations, present in 10–15% of de novo and up to 25% of therapy-related AML. Thus, we hypothesized that *TP53* aberrations in AML contribute to cell-intrinsic and extrinsic resistance against T-cell-based immunotherapy. Cytotoxicity against *TP53*-deleted (DEL) primary AML cells and *TP53*-knockdown (KD) AML cell lines was reduced in co-cultures with T cells stimulated with the BiTE molecule AMG 330 (CD3×CD33). In addition, T-cell proliferation and proinflammatory cytokine secretion was impaired in co-cultures with *TP53* KD cells. Transwell assays identified the secretome of *TP53* KD AML cells as a key contributor to the immunosuppressive effects. Proteomic analysis revealed TGF-β1 in TP53 KD co-cultures as a mediator of T-cell suppression. RNA sequencing of T cells co-cultured with *TP53* KD cells uncovered a transcriptional shift toward a senescent cell cycle profile. Our data collectively identify the immunosuppressive secretome of *TP53*-deficient AML as a key barrier to T-cell-engaging immunotherapies, underscoring an unmet clinical need for strategies able to restore T-cell function in *TP53* KD AML.

## Introduction

Despite significant therapeutic advances in acute myeloid leukemia (AML), including the approval of targeted agents and epigenetic modulators, targeting *TP53*-mutated (MUT) AML remains a major unmet clinical need. Mutations of *TP53* are found in 10–15% of newly diagnosed AML patients, but prevalence has risen to 25 or 30% in relapsed or therapy-related disease, respectively [1]. These patients are classified as adverse risk according to the 2022 European LeukemiaNet (ELN) risk classification and have a median overall survival (OS) of less than 7 months, irrespective of treatment intensity [2, 3]. First-line treatment therapies for *TP53* MUT AML include induction chemotherapy (i.e., cytarabine + anthracycline or liposomal cytarabine + daunorubicin), or for patients ineligible to receive chemotherapy, hypomethylating agents (HMAs) or venetoclax combined with an HMA (azacitidine) [4]. However, in the VIALE-A trial, patients with *TP53* mutations received no meaningful benefit from venetoclax plus azacitidine (6.0 months median OS) compared to azacitidine alone (5.3 months), underscoring the profound resistance to both intensive and low-intensity regimens [5, 6].

Allogeneic hematopoietic stem cell transplantation (HSCT), the only potentially curative approach available, also fails to overcome this poor prognosis [7]. In a recent CIBMTR analysis, *TP53*-mutated AML was associated with a 2-year OS of 19% and a cumulative relapse incidence exceeding 60%, compared to 45% survival in *TP53*-wild-type AML [8]. Similarly, Lindsley et al. identified *TP53* mutations as the most adverse post-HSCT molecular feature, with a 2-year OS of only 9% [9]. Ineligibility for transplant due to age or comorbidities further limits curative options for many patients.

Beyond conferring chemoresistance, *TP53* mutations have been implicated in shaping an immunosuppressive tumor microenvironment (TME). Preclinical studies have linked loss of *TP53* to altered immune signaling pathways, including reduced expression of antigen presentation machinery and changes in cytokine profiles [10–12]. However, the impact of these changes on T-cell-mediated antileukemic immunity remains poorly defined. As bispecific antibodies and CAR T-cell therapies enter clinical trials in AML, dissecting the immune-modulatory impact of *TP53* aberrations will become more important for patient selection and rational combination strategies.

We hypothesized that genetic aberrations of *TP53* in AML contribute to cell-intrinsic and cell-extrinsic resistance to T-cell-based immunotherapy approaches. To test this, we assessed T-cell function against AML cell lines and primary AML samples (pAML) with or without *TP53* aberrations using the CD3×CD33 BiTE molecule AMG 330. We found that T-cell activity is impaired upon co-culture with *TP53* knockdown AML cells and identified their secretome as a central mediator of this effect. Finally, we show that secretion of transforming growth factor-β1 (TGF-β1) by *TP53* aberrant cells induces T-cell cycle arrest, resulting in reduced proliferation and diminished cytotoxic capacity.

## Methods

### Cell lines and cell culture

MV4-11 *TP53* knockdown (KD) and wild-type (WT), MOLM-13 *TP53* KD and WT along with OCI-AML3 *TP53* KD and WT were provided by Michael Andreeff (MD Anderson Cancer Center, Houston, TX). *TP53* KD was achieved using a lentiviral vector and short hairpin RNA (shRNA) [13]. Cell lines were routinely tested for mycoplasm contamination. MV4-11 *TP53* WT and KD cells were authenticated in 2023, respectively 2020 at the DKFZ via STR.

### Human primary biological material

T cells from healthy donors (HDs) and AML patients were isolated from peripheral blood mononuclear cells (PBMCs) using a PAN T-cell Isolation Kit (Miltenyi Biotec) or the EasySep Human CD2 Positive Selection Kit II (Stem Cell), and cultured as previously described [14]. Detailed patient characteristics are summarized in Table S1. All samples were collected with written informed consent in accordance with the Declaration of Helsinki and approval by the Institutional Review Board of the LMU Munich.

### T-cell-mediated cytotoxicity and proliferation assays

T cells from HDs or pAML patients were co-cultured with *TP53* KD or WT AML cell lines or pAML cells at an effector-to-target ratio (E:T) of 1:6 and 5 ng/ml AMG 330 or control T-cell engager (cTCE, specificity against an herbicide). After 3 or 5 days, cells were analyzed using flow cytometry and fluorescently labeled antibodies (Table S2). CD2 was used as surrogate marker to CD3 to identify T-cell populations. Specific lysis was assessed by flow cytometry (Supplementary Fig. 1A) and calculated as follows:

$$\% \,\,{{\rm{specific}}}\,{{\rm{lysis}}}=\left(1{{\mbox{-}}}\frac{{{{{\rm{CD}}}}33}^{+}\,{{{\rm{target}}}}\,{{{\rm{cell}}}}\,{{{\rm{count}}}}\,{{{\rm{AMG}}}}\,330}{{{{{\rm{CD}}}}33}^{+}\,{{{\rm{target}}}}\,{{{\rm{cell}}}}\,{{{\rm{count}}}}\,{{{\rm{cTCE}}}}}\right) \times 100.$$

T-cell proliferation was evaluated using the CellTrace Far Red Proliferation Kit (Thermo Fisher Scientific) (Supplementary Fig. 1C) or by calculating the fold change of absolute T-cell counts as follows:

$${{\rm{T}}}-{{\rm{cell}}}\,{{\rm{foldchange}}}=\left(\frac{{{{{\rm{CD}}}}2}^{+}\,{{{\rm{cell}}}}\,{{{\rm{count}}}}\,{{{\rm{readout}}}}\,{{{\rm{day}}}}}{ {{{{\rm{CD}}}}2}^{+}\,{{{\rm{cell}}}}\,{{{\rm{count}}}}\,{{{\rm{day}}}}\,0}\right).$$

### Bulk RNA sequencing

In total, 5 × 10^5 ^T cells were sorted from 4-day co-cultures with *TP53* KD and WT (E:T 1:6) and lysed in Trizol (Sigma–Aldrich). Total RNA was extracted using the RNA Clean and Concentrator-25 Kit (Zymo Research). RNA sequencing was performed using the prime-seq method developed by Janjic et al. [15] and improved by Pförtner et al. (2025) [15, 16]. The complete protocol for prime-seq, along with the primer sequences, can be accessed at protocols.io (https://www.protocols.io/view/prime-seq-2-14egn97kpl5d/v1). Sequencing was conducted on an Illumina NextSeq2000 instrument, configured with the following parameters: read 1 (28 bases), read 2 (8 bases), read 3 (8 bases), and read 4 (93 bases). Quality assessment of the fastq data files was conducted using fastqc (v 0.11.5) [17]. Read counts per gene were performed with Star (version 2.7.6a) using the default parameters. Expression was calculated with RSEM (1.3.3) and is shown in transcripts per million (TPM). Differential expression was estimated with DSeq2 (1.28.1). Heatmaps are based on log_2_-transformed TPM values. Pathway enrichment analysis was based on the human MSigDB Collection v5p2 and was performed on ranked test statistics with the Bioconductor package “fgsea” (version 1.14.0). Volcano plots were created using the “EnhancedVolcano” plot in R. Genes for Gene Ontology (GO) analysis were selected using *p* < 0.01 and log_2_foldchange > 1 (upregulated) or <−1 (downregulated). To identify biological pathways enriched in differentially expressed genes (DEGs), we performed gene set enrichment analysis (GSEA) using the clusterProfiler package in R (v4.6.0). GO enrichment analysis was carried out using the gseGO() function. HALLMARK gene sets, curated by the Molecular Signatures Database (MSigDB), were retrieved using the msigdbr package. Gene symbols from the ranked list were matched with HALLMARK gene set annotations to create the input for GSEA.

### In vivo studies

All experiments were approved and performed in accordance with the guidelines implemented by the Regierung von Oberbayern. A sample size of *n* = 6 was chosen based on previous studies using comparable mouse models. In vivo Imaging System Platform Lumina X5 (IVIS) was used to measure the BLI signal. Animals were randomized into treatment groups according to BLI signal to ensure equal distribution of tumor spread upon T cell and treatment injection. Investigators were blinded to allocation during experiments and outcome assessment, and two different investigators performed the mouse treatment and subsequent analysis. 7-week old female NOD-Prkdc^scid^-IL2rg^Tm1^/Rj (NXG) mice were engrafted intravenously (i.v.) with 1×10^6^ MV4-11^LUC GFP^ *TP53* KD or WT cells. After 7 days, 5 × 10^6^ HD T cells were injected i.v. Mice were treated with 100 µg/kg AMG 673 or cTCE every 7 days. Tumor growth was monitored using in vivo bioluminescence imaging and health status was checked every other day. Peripheral blood was collected on day 19 after injection of AML cells. The experiment was terminated on day 23 and bone marrow, spleen, and blood were harvested.

### Statistics

Statistical analysis was performed using GraphPad Prism 10 for macOS (version 10.5.0) applying the two-tailed Student´s *t*test for comparison of two groups. Comparisons of data from more than two groups were performed using one-way analysis of variance (ANOVA) testing followed by a Śidák´s multiple comparison test. Due to sample size, normal distribution was assumed. For all comparisons, statistical significance was considered if *p* < 0.05. Unless indicated otherwise, experiments were conducted with a sample size of *n* = 6 (biological replicates) and replicated two times each. Outliers were identified and excluded using the ROUT method.

## Results

### Primary AML samples with *TP53* deletion suppress T-cell function in an autologous and allogeneic setting

**Fig. 1:**
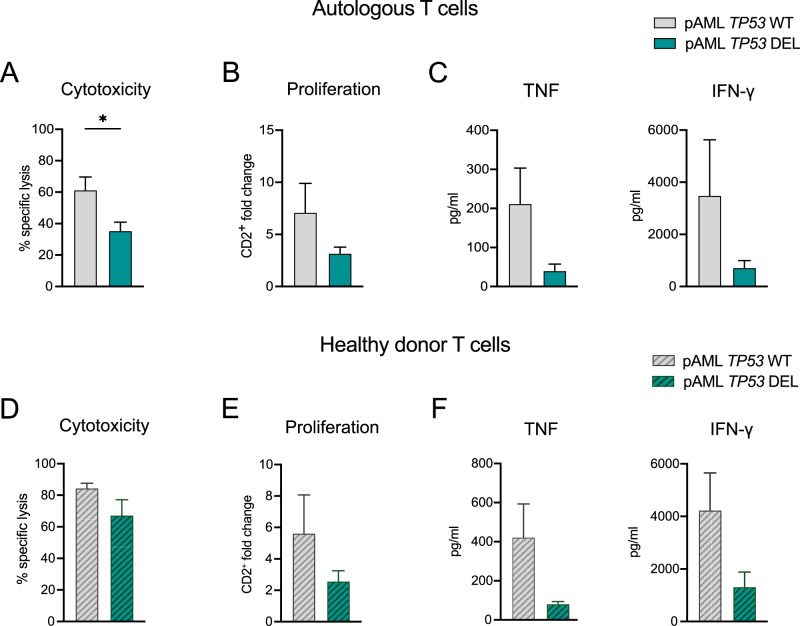
Primary AML samples with TP53 deletion suppress T-cell function in autologous and allogeneic settings. **A** AMG 330-mediated cytotoxicity of primary AML cells (pAML) from patients with or without *TP53* aberration on day 5 (*n* = 8). T cells were isolated by positive selection and co-cultured with pAML cells from the same patient. **B** T-cell proliferation determined via CD2^+^ fold change (*n* = 5) in corresponding co-culture (day5/day0). **C** Secretion of TNF and IFN-γ (*n* = 5) after corresponding co-culture with pAML *TP53* DEL or *TP53* WT, detected by cytometric bead array. **D** AMG 330-mediated cytotoxicity (*n* = 11) of HD T cells against pAML cells with or without *TP53* deletion on day 5. **E** HD T-cell proliferation determined via CD2^+^ fold change (*n* = 11) of corresponding co-culture (day 5/day 0). **F** Secretion of TNF and IFN-γ (n = 11) after co-culture detected by cytometric bead array. All experiments were performed at an E:T ratio of 1:6 with 5 ng/ml AMG 330 or cTCE. All graphs present mean + SEM values. Statistical analysis: paired (**A-E**) or unpaired (**C**–TNF) *t-*test; *p < 0.05.

To assess the impact of *TP53* deletions on T-cell-based immunotherapy, we compared T-cell function against *TP53* DEL and WT pAML cells obtained at initial diagnosis. First, an autologous co-culture system was used in which T cells from AML patients (Table S1) were tested against their matched pAML cells obtained from *TP53* DEL or WT cases. Co-cultures were set up for 5 days at an E:T ratio of 1:6 in the presence of AMG 330 or cTCE. We observed a decrease in AMG 330-mediated cytotoxicity in *TP53* DEL compared to *TP53* WT co-cultures (Fig. 1A). This was accompanied by a trend for lower T-cell proliferation and decreased secretion of the proinflammatory cytokines TNF and IFN-γ (Fig. 1B, C). To further assess the effects of *TP53* DEL AML cells on T-cell function, while excluding any intrinsic impact of *TP53* aberrations within the pAML-derived T cells, we next co-cultured *TP53* DEL and *TP53* WT pAML cells with healthy donor (HD) T cells. In line with the results from the autologous setup, we observed reduced specific lysis along with a reduction in HD T-cell proliferation and secretion of TNF and IFN-γ in *TP53* DEL co-cultures (Fig. 1D–F). Together, these data indicate that *TP53* DEL pAML cells induce T-cell dysfunction in T cells derived from patients and HDs alike.

### AMG 330-mediated cytotoxicity and T-cell function are reduced upon co-culture with MV4-11 *TP53* KD

Next, we aimed to establish an in vitro system that would reproducibly mimic the effects observed in pAML samples. Hence, we used the AML cell lines MV4-11, OCI-AML3, and MOLM-13 harboring a stable shRNA-mediated *TP53* KD (Supplementary Fig. S2A). All three *TP53* KD cell lines showed CD33 expression levels in range with aberrant *TP53* in AML cell lines (Supplementary Fig. S2B).

**Fig. 2:**
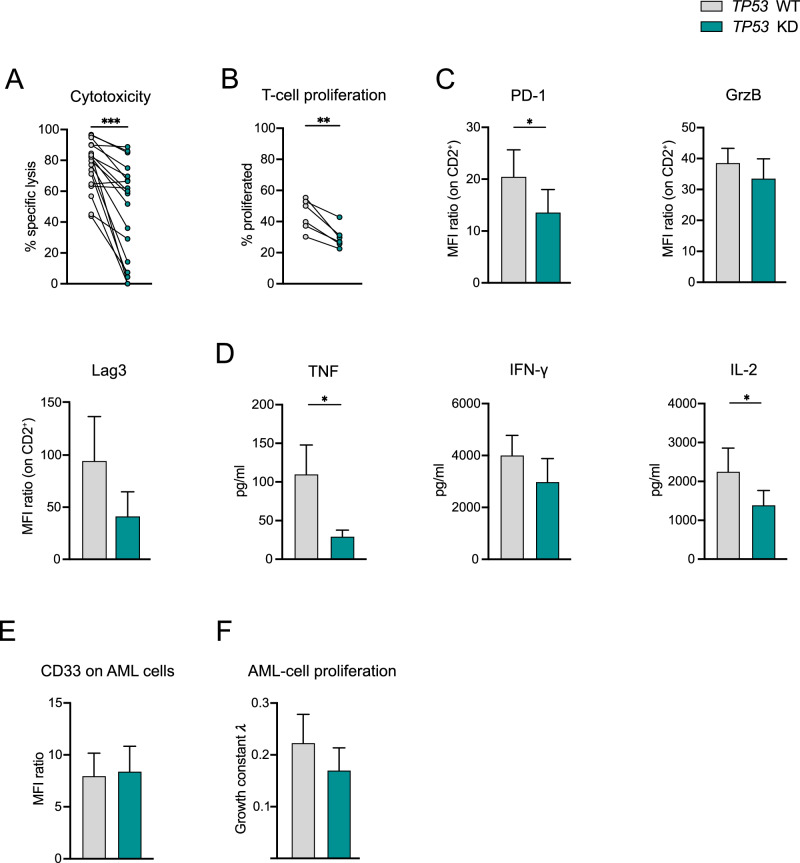
AMG 330-mediated cytotoxicity and T-cell function is reduced upon co-culture with MV4-11 *TP53* KD compared to *TP53* WT. AMG 330-mediated cytotoxicity (*n* = 18, **A**) and proliferation (*n* = 6, **B**) of HD T cells co-cultured with the AML cell line MV4-11 *TP53* KD or WT for 3 days at an E:T ratio of 1:6 and with 5 ng/ml AMG 330 or cTCE. HD T-cell proliferation was assessed with FarRed staining and based on the cTCE condition. **C** Activation and exhaustion-associated surface marker expression on T cells in respective co-cultures. MFI ratio was calculated based on the corresponding isotype control (*n* = 6–7). **D** Secretion of TNF, IFN-γ, and IL-2 on day 3 of co-culture, determined by cytometric bead array (*n* = 8). **E** CD33 target antigen expression on MV4-11 *TP53* KD and WT AML cells determined under baseline conditions by surface marker expression using flow cytometry (*n* = 6). **F** Proliferative capacity of *TP53* KD and WT was determined by calculating the growth constant λ based on the proliferation from day 0 to day 3 (*n* = 5). All graphs present mean + SEM values. Statistical analysis: paired (**A**–**D**) or unpaired (**E**, **F**) *t*-test; *p < 0.05, **p < 0.01, ***p < 0.0001.

To study the impact of *TP53* KD on T-cell-based immunotherapy, MV4-11, MOLM-13, and OCI-AML3 *TP53* KD cell lines or their WT counterparts were co-cultured with HD T cells in the presence of AMG 330. In line with the data using pAML, AMG 330-mediated cytotoxicity was significantly lower against MV4-11 *TP53* KD cells than against WT on days 3 (Fig. 2A) and 5 (Supplementary Fig. S2C) of co-culture. Similar trends were observed for MOLM-13 and OCI-AML3 (Supplementary Fig. S2D-E). Additionally, HD T-cell proliferation was decreased in co-cultures with *TP53* KD compared to *TP53* WT (Fig. 2B, Supplementary Fig. S2C–E). Given that the greatest differences between *TP53* WT and KD were observed with MV4-11 cells, we selected this cell line for further analysis (Supplementary Fig. S2C–E). In a 3-day co-culture with MV4-11 *TP53* KD, T cells showed lower expression of the activation-associated molecules PD-1, GrzB, and Lag3 (Fig. 2C). In line with this, we observed a distinct decrease in the secretion of the proinflammatory cytokines IFN-γ, TNF, and IL-2 into the supernatants of *TP53* KD co-cultures (Fig. 2D). Next, we excluded possible differences in baseline CD33 surface expression and growth kinetics between *TP53* KD and WT (Fig. 2E, F). We then asked whether our previous observations were limited to the use of BiTE molecules. Co-cultures with anti-CD33 CAR T cells were set up and showed reduced specific lysis of *TP53* KD compared to *TP53* WT AML cell lines, except for OCI-AML3, for which no differences were detected (Supplementary Fig. S3A–C). We then assessed whether *TP53* KD mainly affected CD4^+^ or CD8^+^ T cells by performing cytotoxicity assays using AMG 330 and either CD4^+^ or CD8^+^ isolated T-cell populations or CD3^+^ pan cells as controls. Interestingly, the effect appeared additive across CD4^+^ or CD8^+^ T cells (Supplementary Fig. S4A–E). Overall, our in vitro system consistently demonstrated reduced T-cell function upon AMG 330-mediated cytotoxicity of *TP53*-KD AML, mirroring the findings in pAML.

### *TP53* KD-induced metabolic shifts alter AML but not T-cell fitness

Given the impaired T-cell proliferation and cytotoxicity observed in co-cultures with *TP53* KD AML cells, we next sought to identify *TP53* KD-related molecular signatures of this mediated suppressive effect on T-cell function. We focused on three candidate pathways: altered expression of costimulatory or coinhibitory surface molecules, differences in metabolic activity, and transcriptional changes in AML cells.

**Fig. 3:**
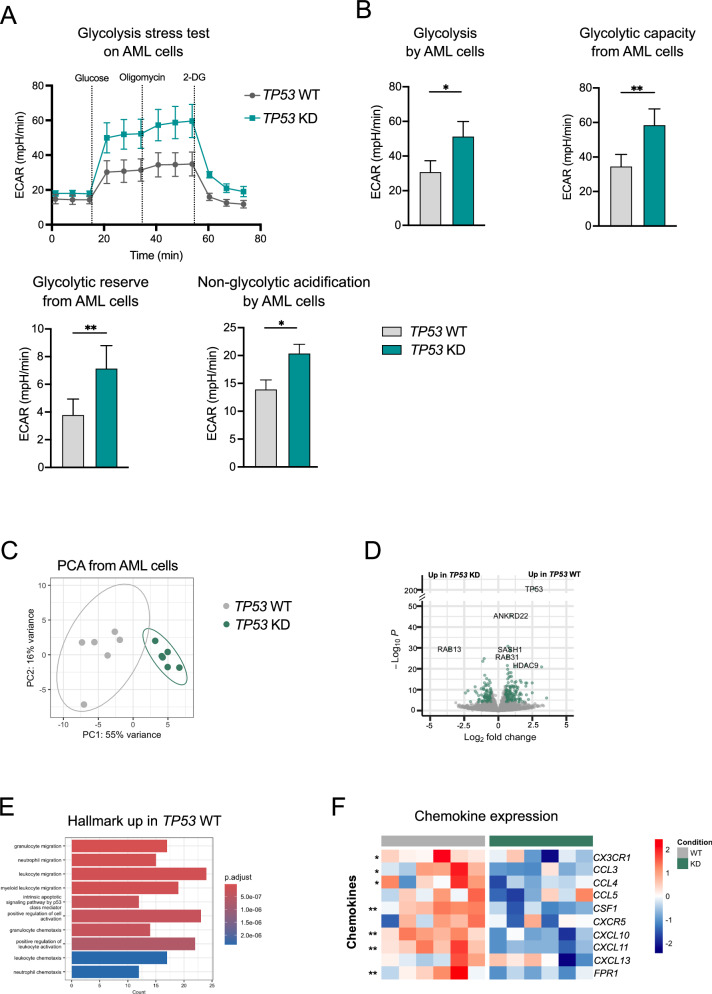
*TP53* KD-induced metabolic shifts alter AML but not T-cell fitness. **A** Kinetic plot of normalized extracellular acidification rate (ECAR) during a glycolysis stress test of MV4-11 *TP53* KD and WT AML cells after 3 days of co-culture with HD T cells (E:T 1:6; 5 ng/ml AMG 330; *n* = 8). **B** Corresponding bar graphs of *TP53* KD and WT after co-culture obtained during glycolysis stress test (*n* = 8). **C** Unbiased principal component analysis of *TP53* KD versus WT after 3-day co-culture with HD T cells (E:T 1:6; 5 ng/ml AMG 330). **D** Corresponding differential gene expression analysis (*n* = 3; *p* < 0.01, FC > 1 or <–1). **E** Pathway analysis of hallmark gene sets upregulated in *TP53* WT AML cells upon co-culture. **F** Clustering of chemokine expression in *TP53* KD and WT AML cells from respective co-cultures. ECAR = extracellular acidification rate. All graphs present mean + SEM values. Statistical analysis: paired *t-*test (**B**); *p < 0.05, **p < 0.01.

To assess the first, we measured CD80, CD86, OX40L, and PD-L1 on MV4-11 *TP53* KD and WT cells at baseline and after IFN-γ/TNF-α stimulation, mimicking exposure by AMG 330-activated T cells. However, no expression differences were observed, suggesting a limited role in *TP53*-driven immune evasion (Supplemental Fig. S5A-B). The p53 protein controls the expression of various genes involved in AML cell metabolism and influences the axis between glycolysis and oxidative phosphorylation [10, 18]. We therefore tested whether treatment with AMG 330 would induce changes in metabolic fitness. To do so, cytotoxicity assays with MV4-11 *TP53* KD or WT cells and HD T cells were performed. After three days, AML cells were isolated and tested for their metabolic profiles in Seahorse assays. Indeed, *TP53* KD AML cells showed increased glycolytic activity after co-culture (Fig. 3A). Furthermore, *TP53* KD cells maintained higher glycolytic capacity, a higher glycolytic reserve, and showed higher non-glycolytic acidification (Fig. 3B). Interestingly, differences in oxidative phosphorylation between *TP53* KD and WT cells were not as pronounced (Supplementary Fig. S5C-D). However, although the altered metabolic profile of the *TP53* KD AML cells might change their metabolic fitness, it seems unlikely that this solely accounts for their ability to impair T-cell function.

Hence, to identify transcriptional changes in the molecular signature of the AML cells, we performed bulk RNA sequencing (RNA-seq) on *TP53* KD and WT after a 3-day co-culture with HD T cells. Unbiased principal component analysis revealed clustering of *TP53* KD and WT, respectively (Fig. 3C). Differential gene expression analysis identified 78 significantly upregulated genes and 116 significantly downregulated genes in *TP53* WT vs. KD (Fig. 3D). *TP53* KD cells showed reduced inflammatory cytokine and immune chemotactic gene expression, suggesting an altered secretome (Fig. 3D–F, Supplementary Fig. 5E). Although metabolic shifts might enhance *TP53* KD AML cell fitness, RNA-seq data indicated that their secretomes modulate T-cell activity.

### The secretome of *TP53* KD AML cells impairs T-cell function

**Fig. 4:**
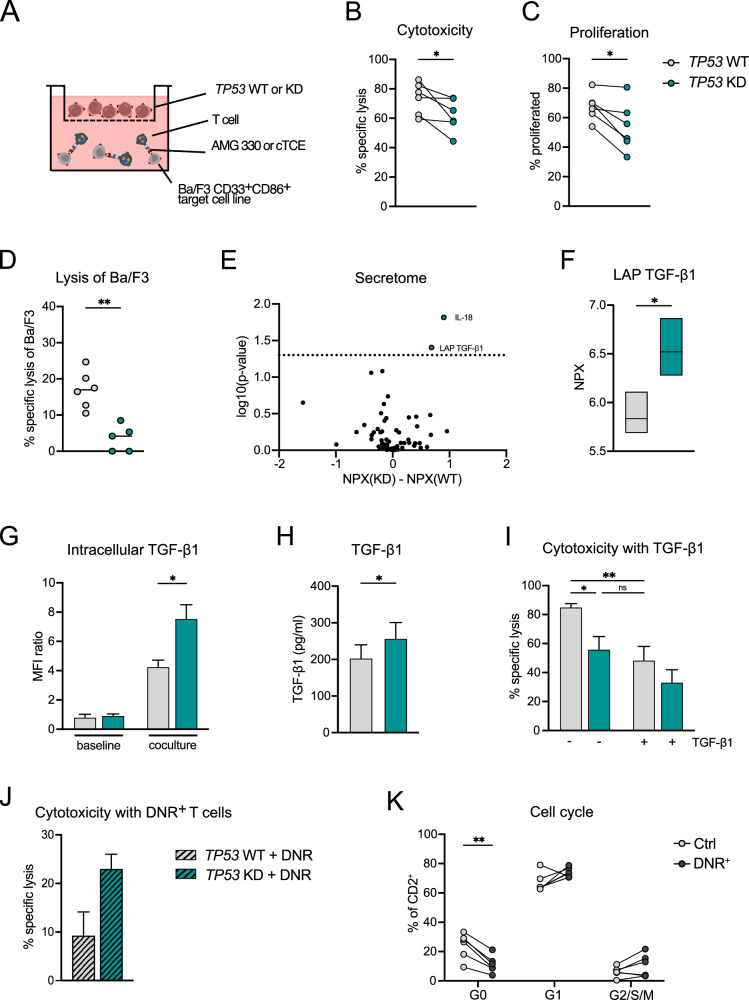
The secretome of *TP53* KD AML cells impairs T-cell function. **A** Experimental setup of the transwell assay. MV4-11 *TP53* KD or WT AML cells were added to the upper chamber; HD T cells, AMG 330 or cTCE, and Ba/F3 CD33^+^CD86^+^ target cells were added to the lower chamber. **B**, **C** AMG 330-mediated cytotoxicity and proliferation of HD T cells under the influence of the secretome of *TP53* KD or WT. T-cell proliferation was determined with FarRed staining and based on the cTCE condition (*n* = 6). **D** Co-culture of Ba/F3 CD33^+^CD86^+^ cells with HD T cells, AMG 330, or cTCE and conditioned medium from a previous co-culture of *TP53* KD or WT with HD T cells and AMG 330 (*n* = 6, one value did not pass the outlier test). **E** Olink analysis of the co-culture supernatant from *TP53* KD or WT with HD T cells. Green data points indicate significant differences between WT and KD. **F** Boxplot of LAP TGF-β1 of Olink® analysis (*n* = 3). **G** Intracellular expression of TGF-β1 of *TP53* KD and WT at baseline and after 3-day co-culture. MFI ratio was calculated based on corresponding isotype control (*n* = 3). **H** Secretion of TGF-β1 in co-culture, analyzed by cytometric bead array (*n* = 12). **I** Specific lysis of *TP53* KD or WT AML cells after co-culture with (+) or without (–) addition of 3 ng/µl TGF-β1 on day 0 (*n* = 8). **J** Co-culture of *TP53* KD or WT with DNR-transduced T cells (E:T 1:20, readout on day 3; *n* = 6). **K** Cell cycle analysis of T cells after transduction with a DNR. Cells were stained with 7-AAD and intracellularly with Ki-67 and readout was performed using flow cytometry (*n* = 6). NPX = normalized protein expression. DNR = double negative receptor. All experiments were performed for 3 days at an E:T ratio of 1:6 and with 5 ng/ml AMG 330 or cTCE. Graphs present the mean + SEM values. Statistical analysis: paired (**B**, **C**) or unpaired (**D**, **F**–**H**) *t-*test or two-way ANOVA with Šidák´s multiple comparison test (**I**–**K**); *ns p* > 0.05, *p < 0.05, **p < 0.01, ***p < 0.0001.

Next, we analyzed the impact of the *TP53* KD secretome on T cells using transwell assays. *TP53* KD or WT cells were cultured in the upper chamber, preventing cell–cell contact with T cells while allowing solutes to cross the membrane. Simultaneously, T cells were cultured in the lower chamber with AMG 330 or cTCE and the model cell line Ba/F3 expressing CD33 and CD86 (Fig. 4A). Interestingly, the suppressive effect of *TP53* KD AML cells on AMG 330-mediated cytotoxicity and HD T-cell proliferation was maintained in these transwell assays, indicating that soluble factors contribute to impairment of T-cell function (Fig. 4B, C). To validate this, we cultured Ba/F3 CD33^+^CD86^+^ target cells with T cells in conditioned medium from *TP53* KD or WT co-cultures and observed reduced specific lysis in the *TP53* KD condition (Fig. 4D). A multiplexed proteomic analysis of secreted proteins (Olink) after a 3-day co-culture of MV4-11 *TP53* KD or WT with HD T cells revealed significantly greater secretion of IL-18 and LAP (latency-associated peptide)-TGF-β1 into the supernatants of *TP53* KD co-cultures (Fig. 4E, F). However, ELISAs performed subsequently could not confirm the differential expression of IL-18 observed in the proteomic screen (Supplementary Fig. S6A-B). Furthermore, addition of IL-18 to the co-culture with *TP53* WT impaired neither lysis nor T-cell proliferation (Supplementary Fig. S5C-D), implying that IL-18 is unlikely to contribute to the effects of *TP53* KD on T cells. We then evaluated TGF-β1 as a candidate immunomodulator. *TP53* KD co-cultures showed increased secretion of LAP-TGF-β1. Focusing on the active form [19], we aimed to verify if TGF-β1 would be secreted by AML cells and therefore measured intracellular levels. Interestingly, while only low levels of intracellular *TGF-β1* were detected at baseline, we measured higher levels in *TP53* KD compared to WT cells upon co-culture (Fig. 4G). Furthermore, cytometric bead arrays of the co-culture supernatants revealed higher levels of secreted TGF-β1 in the *TP53* KD condition (Fig. 4H), confirming the secretome results. To test the impact of TGF-β1 on T cells, we added recombinant TGF-β1 to the co-culture, which decreased the specific lysis of *TP53* WT significantly to levels comparable with *TP53* KD (Fig. 4I). To link the observed dysfunction of T cells with increased levels of TGF-β1, we tried to rescue the effect by blocking the corresponding receptor on T cells. T cells were transduced with a dominant negative receptor (DNR), lacking the intracellular motif required for TGF-β1-mediated signal transduction [20]. Upon transduction, the reduced lysis in the *TP53* KD cells that was previously observed was abolished (Fig. 4J). Moreover, TGF-β1 is known to alter T-cell function by reducing their T-cell proliferation [21]. We therefore performed a cell-cycle analysis of DNR-transduced T cells and detected a smaller proportion of cells in the resting G0 phase compared to untransduced controls. Concurrently, a greater proportion of DNR^+^ T cells in the G1 as well as G2, S, and mitotic phases was observed (Fig. 4K). Taken together, these results revealed that immune evasion by *TP53* KD AML was mediated by its secretome and that TGF-β1 induced T-cell senescence.

### Transcriptional downregulation of cell cycle-related genes in T cells co-cultured with *TP53* KD AML cells

**Fig. 5:**
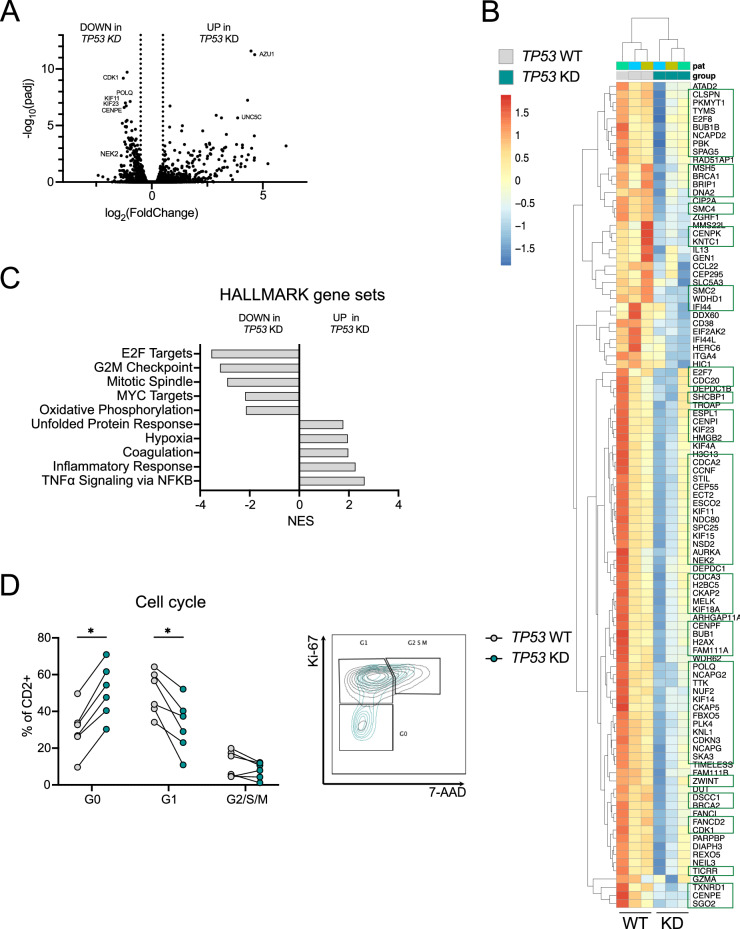
Transcriptional downregulation of cell-cycle-related genes in T cells upon co-culture with *TP53* KD AML cells. **A**–**C** RNA-seq from T cells co-cultured for 4 days with *TP53* KD or WT (E:T 1:6, 5 ng/ml AMG 330). **A** Volcano plot of T cells from corresponding co-culture. *P*_adj_ < 0.05. **B** Pathway analysis of T cells after corresponding co-culture. **C** Heatmap with hierarchical clustering of significantly downregulated genes in T cells after co-culture with *TP53* KD. Green boxes show cell-cycle-related genes. *P*_adj_ < 0.05. **D** Cell cycle analysis of T cells co-cultured with *TP53* KD or WT (E:T 1:6, readout on day 3). Cells were stained with 7-AAD and intracellularly with Ki-67 and readout was performed using flow cytometry. An exemplary contour plot depicts the gating strategy. Gates were set based on the isotype control of Ki-67 (*n* = 6). All graphs present mean + SEM values. Statistical analysis: two-way ANOVA with Šidák´s multiple comparison test (**D**); *p < 0.05.

To evaluate differences in the gene expression profile of T cells co-cultured with *TP53* KD and WT AML cells, we conducted bulk RNA-seq after 3 days. Differential gene expression analysis identified 87 up- and 102 downregulated genes in T cells after co-culture with MV4-11 *TP53* KD compared to WT (Fig. 5A). Interestingly, pathway analysis of T cells co-cultured with *TP53* KD showed downregulation of pathways related to cell-cycle progression: E2F targets, G2M checkpoints, and mitotic spindle formation (Fig. 5C). These findings are consistent with the hierarchical clustering within the heatmap, with genes related to the cell cycle highlighted in green boxes in Fig. 5B and Supplementary Fig. S7. We hypothesized that reduced expression of cell-cycle genes in T cells co-cultured with *TP53* KD impairs T-cell proliferation and lysing capacity. We therefore analyzed the cell-cycle phases of T cells after a co-culture with MV4-11 *TP53* KD or WT cells (Fig. 5D). T cells co-cultured with *TP53* KD remained in a resting and senescent G0 phase, whereas T cells co-cultured with *TP53* WT were predominantly found in G1, ultimately proceeding to mitosis. Overall, these results showed that co-culture of *TP53* KD AML cells impairs T-cell proliferation by driving them towards a senescent state.

### Diminished AMG 673-mediated lysis of *TP53* KD in an in vivo NSG model

**Fig. 6:**
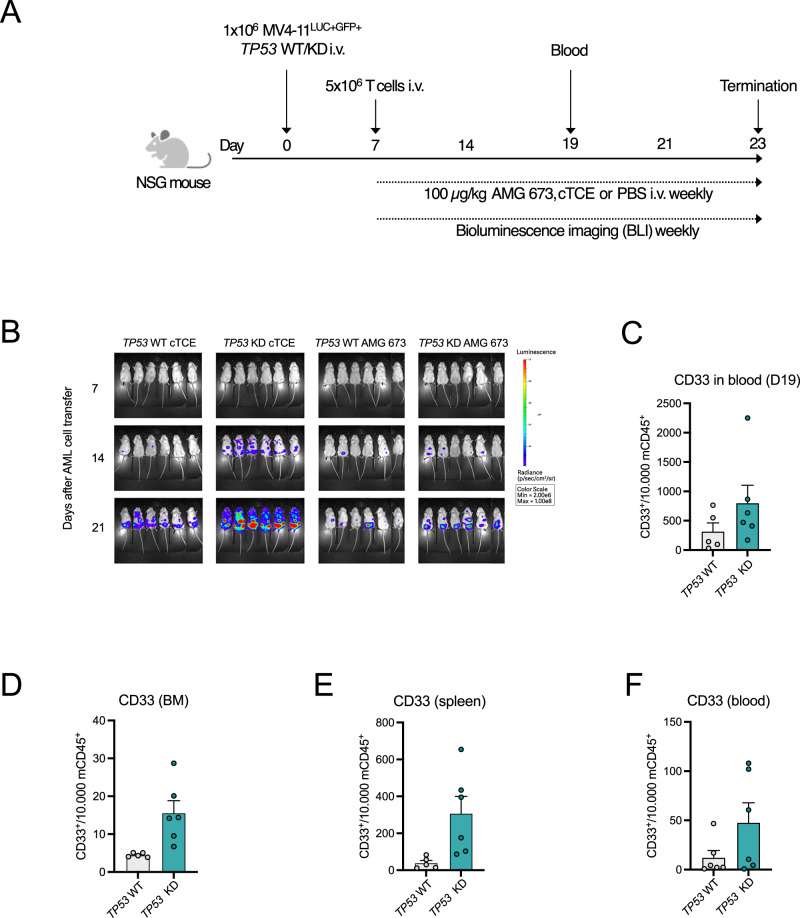
Diminished AMG 673-mediated lysis of *TP53* KD in an in vivo NSG mouse model. **A** Schematic overview of the experimental setup. 1 × 10^6^ MV4-11^LUC+GFP+^ *TP53* KD or WT cells were injected into NSG mice. On day 7, mice were inoculated with 5 × 10^6 ^T cells i.v. together with a first injection of AMG 673 (*n* = 6, 100 µg/kg per injection), cTCE (*n* = 6, 100 µg/kg per injection), or PBS (*n* = 3); AMG 673, cTCE, or PBS were injected i.v. weekly. **B** Bioluminescence imaging was performed weekly. Bioluminescence images from mice on days 7, 14, and 21. **C** CD33^+^ counts from *TP53* KD and WT AMG 673 mice in blood on day 19 (*n* = 5–6, 1 outlier). **D** CD33^+^ counts from *TP53* KD and WT AMG 673 mice in the bone marrow at the day of sacrifice (*n* = 5–6, 1 outlier). **E** CD33^+^ counts from *TP53* KD and WT AMG 673-treated mice in the blood on the day of sacrifice (*n* = 6). **F** CD33^+^ counts from *TP53* KD and WT AMG 673-treated mice in the spleen on the day of sacrifice (*n* = 6). CD33^+^ counts are depicted as counts per 10,000 murine CD45^+^ cells. Graphs present mean + SEM values. Statistical analysis: unpaired *t-*test (**C**–**F**); *p < 0.05.

Lastly, we aimed to confirm our findings in an in vivo experiment. MV4-11^LUC+GFP+^ *TP53* KD or WT cells were engrafted into NSG mice. After 7 days, T cells together with AMG 673 (a half-life-extended CD3×CD33 BiTE molecule), cTCE, or PBS were injected (Fig. 6A). A strong antitumoral response was observed in both *TP53* KD and WT groups treated with AMG 673 compared to cTCE or PBS. Furthermore, *TP53* WT mice showed improved tumor control compared to *TP53* KD (Fig. 6B). These findings were further validated in blood samples taken on day 19 after injection (Fig. 6C). From bioluminescence images acquired on day 21, we concluded that mice in the *TP53* KD cTCE group would soon meet the termination criteria, and the experiment was terminated on day 23. Ex vivo analysis of bone marrow and spleen revealed significantly higher numbers of CD33^+^ cells per 10,000 mCD45^+^ cells in *TP53* KD compared to WT (Fig. 6D, E). These findings were confirmed in the blood (Fig. 6F). Taken together, these data confirmed that both AMG 673-mediated cytotoxicity and T-cell function were reduced with *TP53* KD in this model compared to WT.

## Discussion

Novel T-cell-based immunotherapies, such as T-cell engagers or CAR T cells, have shown high clinical efficacy in various B-cell malignancies [22–24]. However, this success has not yet translated to the myeloid setting [25]. Achieving a mechanistic understanding of resistance to T-cell-based immunotherapy is critical for rational antibody design, patient stratification in clinical trials, and development of next-generation constructs with improved efficacy. Moreover, worse outcomes for *TP53*-mutated compared to *TP53*-wild-type AML patients have been reported across all treatment options, including intensive polychemotherapy, venetoclax–azacytidine, and allo-HSCT [5, 7, 26], underlining the critical role of *TP53* in therapies against AML.

We therefore investigated how *TP53* aberrations in AML affect T-cell function upon treatment with T-cell-based immunotherapies, using the BiTE molecule AMG 330, targeting CD33 and thus a prototypical antigen for AML. In co-cultures with T cells from pAML, *TP53* DEL pAML cells impaired AMG 330-mediated lysis, T-cell proliferation, and cytokine secretion, effects that persisted with HD T cells. Using *TP53* KD AML cell lines, we confirmed reduced AMG 330-mediated lysis of those cells, together with a decrease in HD T-cell proliferation and cytokine secretion. These findings were not limited to treatment with BiTE molecules, but were transferable to a CAR T-cell setting, aligning with recent work by Müller et al., who reported increased CAR T-cell exhaustion along with reduced proliferation and overall function in co-cultures with *TP53*-deficient AML cells [27].

We next asked whether *TP53* KD AML cells impair T-cell-based immunotherapies through the surfaceome or underlying molecular mechanisms. Surface expression of the target antigen CD33 or costimulatory molecules however was comparable, consistent with prior studies showing no differences in the expression of CD33 and PDL1 between *TP53* MUT and WT AML [28, 29]. A role for *TP53* in altering metabolic pathways is well described [18, 30], being mainly associated with an overall higher fitness by increasing the rate of oxidative phosphorylation [31, 32]. Despite a slight increase in oxidative phosphorylation for *TP53* KD in co-culture, the effects were not significant. Instead, co-cultured *TP53* KD AML cells seem to rely more on glycolysis, suggesting a switch toward aerobic glycolysis [10]. These data are consistent with a publication from Harami-Papp et al., describing an increase in glycolysis in *TP53*-mutated breast cancer [33].

Although these results demonstrate an advantage in overall cell fitness of *TP53* KD AML cells, we did not detect alterations in their surfaceome that could explain the impaired T-cell response. Subsequent RNA-seq of *TP53* KD and WT AML cells in co-culture strongly hinted at a profile of reduced chemokine secretion for *TP53* KD cells, providing evidence of an altered secretome. Interestingly, a clinical trial with the CD123×CD3 bispecific antibody flotetuzumab reported upregulation of neutrophil chemoattractants and IFN-inducible molecules in *TP53* MUT AML compared to *TP53* WT samples [34]. However, in our system, RNA-seq of *TP53* KD and WT AML cells upon co-culture indicated a reduction in chemokine secretion in *TP53* KD cells, pointing toward the secretome as a key mediator of immune evasion. This prompted us to investigate the immunomodulatory capacity of the *TP53* KD AML secretome in greater detail. Transwell assays and subsequent proteomic analysis confirmed a critical role of the secretome, mainly TGF-β1, of *TP53* KD cells on T-cell function. A critical role for TGF-β in cancer is well described [35, 36]. TGF-β is known to contribute to immune evasion of cancer cells but also to directly shape the TME [37]. Moreover, TGF-β plays a role in T-cell homeostasis by altering both T-cell proliferation and activation [38], possibly, but not only, by inhibiting IL-2 production [39]. In agreement with this, our data show a reduction of IL-2 secretion in *TP53* KD co-cultures. Additionally, TGF-β has an inhibitory effect on differentiation of naïve T cells [6, 40, 41].

To overcome the effects of TGF-β on immune cells, several therapeutic strategies have been validated in preclinical models, with some progressing to clinical trials. These approaches can be broadly categorized into strategies directly targeting the TGF-β ligand and those inhibiting TGF-β signaling in immune cells by targeting its specific receptors. Ligand traps and TGF-β-neutralizing monoclonal antibodies directly block the ligand, thereby preventing receptor binding and subsequent downstream signaling [42, 43]. For example, Fresolimumab has been tested in a phase I/II clinical trial, among others, in renal cell carcinoma [44]. In addition, several groups used transgenic approaches to inhibit signaling [45]. Gorelik and Flavell used a dnTGF-βRII, demonstrating that inhibiting TGF-β signaling in T cells promotes their expansion, ultimately triggering an immune response capable of eradicating melanoma tumor cells [21]. Our data are consistent with this, showing that inhibition of TGF-β signaling using a DNR in T cells significantly reduced the number of cells in a senescent cell cycle phase as well as having a positive effect on T-cell function.

Lastly, RNA-seq of T cells co-cultured with *TP53* KD AML revealed a shift toward a senescent cell state. Senescence occurs naturally in ageing T cells but can also be induced prematurely in response to cellular stress [27]. Senescent T cells are in cell-cycle arrest, do not proliferate, and show limited killing capacity, but remain viable and metabolically active [46]. Tumor cells are known to actively shape an immunosuppressive TME to evade adaptive immunity. This involves the secretion of suppressive factors such as TGF-β, IL-10 or indoleamine-pyrrole 2,3-dioxygenase, ultimately leading to a recruitment of Tregs [47–51]. It is therefore tempting to speculate that elevated levels of TGF-β in the secretome of *TP53* KD AML treated with AMG 330 drive T cells into a senescent state, impairing their proliferation and killing ability. In line with this hypothesis, Vadakekolathu et al. describe functional enrichment of a senescence-related gene set in TCGA-AML cases harboring *TP53* mutations upon treatment with flotetuzumab [34].

In conclusion, our results highlight that AML-intrinsic genetics critically shape the efficacy of T-cell-based immunotherapies. By demonstrating that *TP53* KD AML cells impair T-cell proliferation through a TGF-β1-dominated suppressive secretome, we identify a novel, cell-intrinsic resistance mechanism that extends beyond previously reported alterations of the AML surfaceome [52]. Although future studies will need to address the spectrum of *TP53* hotspot mutations, our data provide the first evidence that secretome-driven immunosuppression represents a key barrier to effective T-cell engagement.

Elucidating the signaling pathways responsible for enhanced TGF-β secretion in *TP53*-deficient AML might therefore afford new opportunities to restore T-cell function and improve the efficacy of emerging immunotherapies in this high-risk genetic subgroup.

## Supplementary information

Supplemental Material

Supplementary Figure 1

Supplementary Figure 2

Supplementary Figure 3

Supplementary Figure 4

Supplementary Figure 5

Supplementary Figure 6

Supplementary Figure 7

## Data Availability

The RNA-seq data discussed in this article have been deposited in the GEO database under the accession number GSE309028. The datasets generated for this study are available from the corresponding author upon reasonable request.
