# Supplementary material for: TP53 deficiency in AML induces resistance to T-cell engagers through an immunosuppressive secretome
Source: Leukemia. 2026 Jun 1;40(8):1624–35. doi: 10.1038/s41375-026-02991-6 (PMC13421328; doi:10.1038/s41375-026-02991-6)
Supplement: Supplementary file 1 — Supplemental Material [file 41375_2026_2991_MOESM1_ESM.docx]

**Supplementary Material**

**Supplementary Methods**

**Media**

Cell lines were cultured in RPMI 1640 medium (PAN Biotech) containing 10% heat-inactivated fetal bovine serum (FBS, Thermo Fisher Scientific), 0.5 mg/ml penicillin–streptomycin–glutamine (PSG, Thermo Fisher Scientific) and 10 mM HEPES (Carl Roth) at 37°C in a 5% CO_2_ atmosphere. Primary AML cells were precultured for 24 h in α-MEM medium (PAN Biotech) supplemented with 12.5% FBS, 12.5% horse serum (Sigma–Aldrich) and 0.5 mg/ml PSG. The medium over irradiated MS-5 feeder cells was supplemented with recombinant human (rhu) thrombopoietin (Peprotech), rhu IL-3 (Peprotech), rhu granulocyte colony-stimulating factor (Peprotech) and 57.2 μM 2-mercaptoethanol (Sigma–Aldrich) prior to an experiment. MV4-11 *TP53* WT or KD were activated with 5 ng/ml TNF-α (Peprotech) and 500 U/ml IFN-γ (Peprotech) 3 days prior to measurement.

**Flow cytometry**

Functional assays and immunophenotyping were analyzed using flow cytometry (Cytoflex S or LX, Beckmann Coulter). Staining was performed Dulbecco's phosphate-buffered saline (DPBS) containing 1% bovine serum albumin (Miltenyi Biotec) and 2 mM EDTA (Invitrogen) The ratio of the median fluorescence intensity (MFI) of surface and intracellular markers was calculated based on the respective isotype controls (Supplementary Figure S1B):

MFI ratio = $\frac{MFI staining}{MFI isotype control}$.

Living and dead cells were discriminated using the LIVE/DEAD Fixable Aqua Dead Cell Stain Kit (Thermo Fisher Scientific). Intracellular or intranuclear cytokine staining was performed using the BD Cytofix/Cytoperm Kit (BD Biosciences) or the Foxp3/Transcription Factor Staining Buffer set (Thermo Fisher Scientific), respectively, according to the manufacturer's instructions.

**Cytokine protein level quantification**

Supernatants after co-culture were analyzed for IL-2, IL-6, IL-10, tumor necrosis factor (TNF) and interferon-γ (IFN-γ) using the Human Th1/Th2 Cytokine Kit (BD Biosciences) according to the manufacturer’s instructions. TGF-β1 in the co-culture supernatant was measured using the TGF-β1 Single Plex Flex Set (BD Biosciences). Total IL-18 and IL-18BP were measured with the DuoSet ELISA Human Total IL-18 kit (R&D systems) according to the manufacturer's instructions.

# **qPCR**

Total RNA was extracted from *TP53* KD and WT using the RNeasy Mini Kit (Qiagen) according to the manufacturer's instructions. RNA was transcribed using the RevertAid First Strand cDNA Synthesis Kit (Thermo Scientific) together with the RiboLock RNAse Inhibitor (Thermo Scientific). qPCR was performed using the iTaq Universal SYBR Green Supermix (Biorad) and PrimePCR assays for *TGF-β1* (HSA, Biorad) and *TP53* (HSA, Biorad) in triplicate. Relative mRNA expression was calculated based on the cycle threshold (Ct) value and compared to the expression of the housekeeper *ABL-1* (HSA, Biorad).

**Western blotting**

Proteasomal degradation of p53 in MV4-11 cells was inhibited by adding 10 µM MG132 (Selleck Chemicals) in DMSO to 2×10^6^ cells. DMSO only served as a control. After 4 h, cells were pelleted and washed. Next, cells were lysed using RIPA buffer, and the lysate was fractionated by SDS-PAGE. Proteins were transferred to a PVDF membrane using the Mini-PROTEAN Tetra Cell electrophoresis chamber according to the manufacturer's instructions. The membrane was blocked in 5% milk powder in TBS-T for 1 h and subsequently incubated with the primary anti-p53 antibody (Santa Cruz Biotechnology) at 4°C for 12 h. Afterwards, three washing steps in TBS-T were followed by incubation for 1 h with horseradish peroxidase (HRP)-coupled anti-mouse antibody (IgG_Κ_, Santa Cruz Biotechnology) as a secondary antibody. The membrane was washed three times before the HRP substrate (Sigma–Aldrich) was added. The chemiluminescent reaction was evaluated using the Amersham Imager 600.

**Glucose uptake assays**

5×10^5^ MV4-11 *TP53* KD and WT cells were incubated with 0, 50, 100 and 200 µM 2-[*N*-(7-nitrobenz-2-oxa-1,3-diazol-4-yl) amino]-2-deoxyglucose (2-NBDG, Cayman Chemical) for 2 h at 37°C in glucose-free medium. Cells were pelleted, washed and analyzed using flow cytometry^1^.

**Growth constant determination**

To determine the growth constant λ, MV4-11 *TP53* KD and WT cells were cultivated for 3 days and counted on days 0 and 3 using flow cytometry. Assuming exponential growth, λ was calculated as follows^2^:

$$\lambda=\frac{\ln\left( N_{t} \right)-ln(N_{0})}{t-t_{0}}$$

where *N_t_* = cell count on day 3, *N*_0_ = cell count on day 0, *t* = observation time, and *t*_0_ = initial time.

**Seahorse cell metabolic stress tests**

*TP53* KD and WT AML cells were co-cultured with healthy donor (HD) T cells at an E:T ratio of 1:6 together with 5 ng/ml AMG 330. After 3 days, AML cells were negatively isolated and seeded at a density of 2.5×10^5^ cells per well in triplicate on poly-D-lysine-coated Seahorse plates (Santa Cruz Biotechnology). Steady-state MV4-11 *TP53* KD or WT cells served as controls. The oxygen consumption rate (OCR, pmol/min) was measured using the appropriate kits (Agilent) at baseline and after injection of oligomycin (1 μM), carbonyl cyanide-*p*-trifluoromethoxyphenylhydrazone (FCCP, 1 μM), and antimycin A/rotenone (1 μM). The extracellular acidification rate (ECAR, pmol/min) was measured using the appropriate kit (Agilent, 103020-100) with added glucose (10 mM), oligomycin (1 µM) and 2-deoxy-D-glucose (2-DG, 50 mM). Measurements were performed on a Seahorse XFe96 analyzer. Glycolytic reserve was calculated by subtracting the glycolytic rate from the glycolytic capacity. ATP-linked respiration was calculated by subtracting the proton leak from the basal respiration. The spare capacity was calculated by subtracting the basal respiration from the maximal respiration.

**Transwell assays**

The impact of the secretome was assessed using 24-well transwell inlets with a 3 µm pore membrane (Greiner). A total of 1.5×10^6^ *TP53* KD or WT cells were plated in the upper chambers of the plates, and 9×10^5^ Ba/F3 CD33^+^CD86^+^ target cells were co-cultured with 9×10^4^ HD T cells in the presence of 5 ng/ml AMG 330 or cTCE in the lower chambers. After 3 days, T-cell proliferation and specific lysis of target cells were analyzed by flow cytometry.

**Proteomic analysis (Olink)**

The proteomes of the co-culture supernatants were analyzed using the Olink platform and the Target 96 Immuno-Oncology Panel (Olink Bioscience). In brief, MV4-11 *TP53* KD and WT cells were co-cultured with HD T cells and 5 ng/ml AMG 330. After 3 days, cells were centrifuged (550 rcf, 5 min, 20°C) and the supernatants collected. Analysis was performed at the Metabolomics and Proteomics Core Facility of the Helmholtz Centre, German Research Centre for Environmental Health (Neuherberg, Germany). Measured protein concentrations were expressed as normalized protein expression (NPX) units.

**DNR T-cell manufacture**

T cells from HDs were isolated as previously described and cultured in TexMACS medium supplemented with 10 ng/ml IL-7 and 5 ng/ml IL-15. T cells were activated for 48 h using human TransAct (1:100). The producer cell line for retroviral transduction of the double negative receptor (DNR) was kindly provided by Sebastian Kobold (LMU Munich, Munich). Transduction was performed for 90 minutes at 32°C on retronectin-coated plates (15 µg/ml) and with two consecutive hits.

**CAR T cell production**

Human T cells were isolated using EasySep Human T Cell Isolation Kit (Stemcell) according to the manufacturers’ instructions. The cells were activated at a concentration of 1 × 106 cells/ml with 1:100 TransAct (Miltenyi Biotec) and cultured in Human TexMACS (Miltenyi Biotec) + 1% pen–strep–L-glutamine and 10 ng/ml IL-7/IL-15 (Peprotech) for 48 hours. Non tissue culture-treated plates were treated for 24 hours at 4 °C with retronectin (Takarabio) according to manufacturers’ instructions. Retrovirus was harvested, centrifuged and filtered, from the supernatant of a virus producer cell line (cultured in D10) containing a sequence encoding for the anti-CD33-CD28-CD3z CAR. The retroviral producer cell line 293Vec‑RD114‑CAR‑CD33 was kindly provided by Sebastian Kobold (LMU Munich, Munich)^3^. The retroviral particles were spun onto the retronectin coated plates (3000 g, 32 °C) for 1.5 hours. The supernatant was discarded, and the activated T cells were added. The CAR T cells were kept at a density of 1 × 106 cells/ml and expanded for up to 14 days. The transduction efficiency was determined by c-myc marker gene expression and subsequent flow cytometric analysis.

**Patient characteristics**

*TP53*-deleted patients had a median age of 69.5 years at first diagnosis. 70% (n=10) of the patients were male. AML control patients without *TP53* aberrations had a median age of 68 years; 53.3% were male (n=16).

**Tables**

**Table S1: Characteristics of AML patients used for in vitro analysis.**

| Patient | Diagnosis | Sex | Age | Blast (%) | *TP53* status | Karyotype |
| --- | --- | --- | --- | --- | --- | --- |
| 1 | FD | m | 53 | 17% | *TP53* DEL | complex |
| 2 | FD | m | 70 | 27% | *TP53* DEL | complex |
| 3 | FD | m | 55 | 84% | *TP53* DEL + monosomy 17 | complex |
| 4 | FD | f | 78 | 80% | *TP53* DEL | aberrant |
| 5 | FD | f | 73 | 82% | *TP53* DEL | aberrant, complex |
| 6 | FD | f | 71 | 80% | *TP53* DEL | complex |
| 7 | FD | m | 34 | 64% | *TP53* DEL | aberrant, complex |
| 8 | FD | m | 69 | 86% | *TP53* DEL | aberrant, complex |
| 9 | FD | m | 77 | 77% | *TP53* DEL | aberrant |
| 10 | FD | m | 25 | 85% | *TP53* DEL | aberrant |
| 11 | FD | f | 74 | 70% | *TP53* WT | normal |
| 12 | FD | m | 75 | 67% | *TP53* WT | aberrant, complex, AN |
| 13 | FD | m | 80 | 33% | *TP53* WT | normal |
| 14 | FD | f | 42 | 16% | *TP53* WT | aberrant/AN |
| 15 | FD | m | 57 | 21% | *TP53* WT | normal |
| 16 | FD | m | 73 | 64% | *TP53* WT | aberrant |
| 17 | FD | f | 53 | 81% | *TP53* WT | normal |
| 18 | FD | m | 69 | 88% | *TP53* WT | normal |
| 19 | FD | f | 54 | 3% | *TP53* WT | normal |
| 20 | FD | m | 45 | 16% | *TP53* WT | normal |
| 21 | FD | m | 61 | 80% | *TP53* WT | aberrant |
| 22 | FD | m | 68 | 80% | *TP53* WT | AN |
| 23 | FD | m | 83 | kA | *TP53* WT | aberrant |
| 24 | FD | m | 72 | 78% | *TP53* WT | - |
| 25 | FD | f | 31 | 6% | *TP53* WT | normal |
| 26 | FD | f | 45 | 81% | *TP53* WT | normal |

FD: first diagnosis, m: male, f: female, DEL: deleted, WT: wild-type, AN: aberrant and normal cells

**Table S2: Antibodies used for flow cytometry.**

| **Antigen (human)** | **Fluorochrome** | **Isotype** | **Manufacturer** | **Cat. #** | **Clone** |
| --- | --- | --- | --- | --- | --- |
| CD2 | PE  APC  FITC  BV605 | Mouse IgG1, κ  Mouse IgG1, κ  Mouse IgG1, κ  Mouse IgG1, κ | Biolegend  Biolegend  Biolegend  Biolegend | 984906  300213  300206  300224 | TS1/8  RPA-2.10  RPA-2.10  RPA-2.10 |
| CD33 | APC  BV421 | Mouse IgG1, κ  Mouse IgG1, κ | Biolegend  Biolegend | 366606  366622 | P67.6  P67.6 |
| CD69 | FITC | Mouse IgG1, κ | Biolegend | 310904 | FN50 |
| CD25 | PE/Dazzle594 | Mouse IgG1, κ | Biolegend | 302646 | BC96 |
| GrB | PE | Mouse IgG1, κ | Biolegend | 372208 | QA16A02 |
| CD223 (Lag-3) | PE Cy7 | Mouse IgG1, κ | Invitrogen | 25-2230-42 | 3DS223H |
| CD366 (Tim3) | PE | Mouse IgG1, κ | Biolegend | 345006 | F38-2E2 |
| CD279 (PD-1) | BV785 | Mouse IgG1, κ | Biolegend | 367432 | NAT105 |
| 4-1BB (CD127) | AF700 | Mouse IgG1, κ | Biolegend | 351344 | A019D5 |
| Ki-67 | BV421 | Mouse IgG1, κ | Biolegend | 350506 | Ki-67 |
| 7-AAD |  |  | Biolegend | 420404 |  |
| CD4 | PerCP CY5.5.  BV785 | Mouse IgG1,κ  Mouse IgG1, κ | Biolegend Biolegend | 300530  300554 | RPA-T4 RPA-T4 |
| CD8 | APC-Cy7  BV421 | Mouse IgG1, κ  Mouse IgG1, κ | Biolegend Biolegend | 344714  344748 | SK1 SK1 |
| CD45RA | PE | Mouse IgG2b, κ | Biolegend | 304108 | HI100 |
| CCR7 | BV650 | Mouse IgG2a, κ | Biolegend | 353234 | G043H7 |
| CD127 | APC Cy7  BV605 | Mouse IgG1, κ  Mouse IgG1, κ | Biolegend  Biolegend | 351348  351334 | A019D5  A019D5 |
| CTLA-4 | BV605 | Mouse IgG2a, κ | Biolegend | 369610 | BNI3 |
| Foxp3 | PE  APC | Mouse IgG1, κ  Mouse IgG1, κ | Biolegend  Invitrogen | 364704  17-4777-42 | QA18A03  236A/E7 |
| IL-10 | PE | Rat IgG1, κ | Biolegend | 501403 | JES3-9D7 |

**Table S3: Reagents.**

| **Name** | **Manufacturer** | **Cat. #** |
| --- | --- | --- |
| 2-Mercaptoethanol | Sigma–Aldrich | 63689 |
| 2-NBDG | Cayman Chemical | 11046 |
| Ammonium persulfate | Sigma–Aldrich | 215589 |
| Alpha MEM Eagle medium | PanBiotech | P04-21500 |
| Brefeldin A | Sigma–Aldrich | B7651-5MG |
| Bromophenol blue | Carl Roth GmbH | A512.1 |
| DPBS | PanBiotech | P04-36500 |
| FcR blocking reagent | Miltenyi Biotec | 130-059-901 |
| Fetal bovine serum | Thermo Fisher Scientific | 10270106 |
| Ficoll Histopaque-1077 Hybri-Max | Sigma–Aldrich | H8889-500ML |
| G-CSF | Peprotech | 300-23 |
| Glycine Pufferan | Carl Roth GmbH | 3908.1 |
| Glycerin | Carl Roth GmbH | 3783.1 |
| HEPES buffer solution (1 M) | Gibco Life Technologies | 15630-056 |
| Horse serum | Sigma–Aldrich | H1270 |
| Horseradish peroxidase (HRP) substrate | Sigma–Aldrich | OR03L |
| HRP substrate | Sigma–Aldrich | WBUKS0100 |
| IFN-γ | Peprotech | 300-02 |
| IL-3 | Peprotech | 200-03 |
| iTaq Universal SYBR Green Supermix | Biorad | 1725121 |
| MACS BSA stock solution | Miltenyi Biotec | 130-091-376 |
| MG132 | Selleck Chemicals | S2619 |
| m-IgGk BP-HRP | Santa Cruz Biotechnology | sc-516102 |
| Milk powder | Carl Roth GmbH | T145.2 |
| Penicillin–streptomycin–L-glutamine, 100X | Thermo Fisher Scientific | 10378016 |
| Pierce RIPA buffer | Thermo Fisher Scientific | 89900 |
| Poly-D-lysine | Santa Cruz Biotechnology | sc-136156 |
| PrimePCR assays for *TGF-β1* (HSA) | Biorad | 10025626 |
| PrimePCR assays for *TP53* (HSA) | Biorad | 10025636 |
| p53 antibody | Santa Cruz Biotechnology | sc-126 |
| RiboLock RNAse Inhibitor | Thermo Fisher Scientific | EO0282 |
| RMPI 1640 | PanBiotech | P04-16500 |
| Rotiphorese NF-acrylamide/bis-solution 30 | Carl Roth GmbH | A124.1 |
| Seahorse XF RPMI, pH 7.4 | Agilent | 103576-100 |
| Seahorse XF, 200 mM glutamine solution | Agilent | 103579-100 |
| SDS | Carl Roth GmbH | 2326.3 |
| Sodium pyruvate (100 mM), 100X | Gibco Life Technologies | 11360-70 |
| TEMED | Carl Roth GmbH | 8142.1 |
| TRIS Pufferan | Carl Roth GmbH | AE15.1 |
| TNF-α | Peprotech | 300-01 |
| TPO | Peprotech | 300-18 |
| TWEEN 20 | Sigma–Aldrich | 11332465001 |
| UltraPureTM 0.5 M EDTA | Invitrogen | 15575-038 |
| RetroNectin® Recombinant Human Fibronectin Fragment | Takarabio | T100A |
| Human TexMACSTM | Miltenyi Biotec | 130-097-196 |
| rh IL-7 | Peprotech | 200-7 |
| rh IL-15 | Peprotech | 200-15 |
| T Cell TransAct™, human | Miltenyi Biotec | 130-111-160 |

**Table S4: Kits.**

| **Kit** | **Manufacturer** | **Cat. #** |
| --- | --- | --- |
| BD Cytofix/Cytoperm Fixation/ Permeabilization Kit | BD Biosciences | 554714 |
| Cholesterol/Cholesterol Ester-Glo Assay | Promega | J3190 |
| DuoSet ELISA Human Total IL-18 | R&D Systems | DY318-05 |
| EasySep Human T Cell Isolation Kit II | Stemcell Technologies | 17883 |
| Foxp3/Transcription Factor Staining Buffer Set | eBioscience | 00-5523-00 |
| Human TGF-β1 Single Plex Set | BD Biosciences | 560429 |
| Human Th1/Th2 Cytokine Kit II | BD Biosciences | 551809 |
| NEBNext Ultra II Directional RNA Library Prep Kit for Illumina | New England BioLabs | E7760 |
| Nuclear Extraction Kit | Abcam | Ab113474 |
| Pan T Cell Isolation Kit | Miltenyi Biotec | 130-096-535 |
| RNA Clean and Concentrator-25 Kit | Zymo Research | R1017 |
| RNeasy Mini Kit | Qiagen | 74104 |
| RevertAid First Strand cDNA Synthesis Kit | Thermo Scientific | K1622 |
| Seahorse XF Cell Mito Stress Test Kit (oligomycin, FCCP, rotenone + antimycin A) | Agilent | 103015-100 |
| Seahorse XF Glycolysis Stress Test Kit (glucose, oligomycin, 2-DG) | Agilent | 103020-100 |
| Seahorse XFe96 FluxPak Mini | Agilent | 102601-100 |
| SREBP-1 Transcription Factor Assay Kit | Abcam | ab133125 |

**Figures**

**Supplementary Figure 1.**

Representative gating strategies for (A) flow-cytometry based cytotoxicity assays, (B) surface marker expression based on MFI ratio against an isotype control, (C) FarRed-based proliferation.

**Supplementary Figure 2.**

(A) Successful *TP53* KD on the protein level was confirmed using western blot analysis. To inhibit proteasomal degradation under baseline conditions, cells were treated with 10 µM MG132 in DMSO. The signal from β-actin served as a loading control. Ctrl = DMSO only. (B) CD33 surface expression by AML cell lines with an endogenous *TP53* aberration and cell lines with an shRNA-mediated *TP53* KD (n=3–6). (C) AMG 330-mediated cytotoxicity (n=6) and proliferation (n=6) of HD T cells co-cultured with MV4-11 *TP53* KD or WT for 5 days (E:T 1:6). (D) AMG-330-mediated cytotoxicity (n=6) and proliferation (n=6) of HD T cells co-cultured with MOLM-13 *TP53* KD or WT for 3 and 5 days (E:T 1:6). (E) AMG-330-mediated cytotoxicity (n=6) and proliferation (n=6) of HD T cells co-cultured with OCI-AML3 *TP53* KD or WT for 3 and 5 days. All graphs present mean ± SEM values. Statistical analysis: paired *t*-test (C-E). *p<0.05, ***p*<0.01, ****p*<0.0001.

**Supplementary Figure 3.**

(A) Specific lysis of MV4-11 *TP53* KD or WT, MOLM-13 *TP53* KD or WT and OCI-AML3 *TP53* KD or WT in co-culture with anti-CD33 CAR T cells (E:T 1:10, readout on day 3). (B) Specific lysis of MV4-11 *TP53* KD or WT, MOLM-13 *TP53* KD or WT and OCI-AML3 *TP53* KD or WT in co-culture with anti-CD33 CAR T cells (E:T 1:20, readout on day 3). (C) Inflammatory cytokine secretion by anti-CD33 CAR T cells upon co-culture with *TP53* KD and WT AML cell lines (E:T 1:20). Specific lysis was calculated based on the respective untransduced T-cell controls. All graphs present mean ± SEM values. Statistical analysis: unpaired (A-B) or paired (C) *t*-test.

**Supplementary Figure 4.**

(A) AMG 330-mediated cytotoxicity (n=6) and (B) proliferation (n=5) of HD T cells co-cultured with MV4-11 *TP53* KD or WT for 3 days (E:T ratio 1:6, 5 ng/ml AMG 330 or cTCE). CD4^+^ and CD8^+^ T cells were positively isolated and mixed in a 1:1 ratio for the CD3^+^ condition. T-cell proliferation was determined with FarRed staining and in comparison to the cTCE condition. (C) Secretion of TNF, IFN-γ and IL-2 by CD3^+^ T cells after corresponding co-culture (n=6). (D) Secretion of TNF, IFN-γ and IL-2 by CD4^+^ T cells after corresponding co-culture (n=6). (E) Secretion of TNF, IFN-γ and IL-2 of CD8^+^ T cells after corresponding co-culture (n=6). All graphs present mean ± SEM values. Statistical analysis: paired (B-E) or unpaired (A) *t*-test; **p*<0.05, ***p*<0.01.

**Supplementary Figure 5.**

(A, B) Antigen expression profiling of markers linked to activation and proliferation along with costimulatory molecules was performed under steady-state conditions and after activation with TNF-α and IFN-γ for 72 h (n=6). (C) Kinetic plot of normalized OCR during a mito stress test of MV4-11 *TP53* KD and WT after 3 days of co-culture with HD T cells (n=8). (D) Corresponding bar graphs for MV4-11 *TP53* KD and WT after co-culture obtained during the mito stress test (n=8). (E) Hallmark pathway analysis of genes upregulated in T cells from a co-culture with *TP53* KD and WT (n=3). OCR = oxygen consumption rate. All experiments were performed at an E:T ratio of 1:6 and with 5 ng/ml AMG 330 or cTCE. HD T-cell proliferation was assessed with FarRed staining and based on the cTCE condition. All graphs present mean ± SEM values. Statistical analysis: paired *t*-test (A, B, D); ***p*<0.01.

**Supplementary Figure 6.**

(A) Total IL-18 and (B) bound IL-18 in the supernatant after a 3-day co-culture with HD T cells, MV4-11 *TP53* KD or WT and AMG 330 determined using ELISA (n=6). (C) AMG 330-mediated cytotoxicity (n=6–8) and (D) proliferation (n=6–8) of HD T cells co-cultured with *TP53* KD or WT with or without further addition of IL-18. The assay was performed for 3 days at an E:T ratio of 1:6. HD T-cell proliferation was calculated based on the fold change of AMG 330 to the cTCE condition. BP = bound protein. All graphs present mean ± SEM values. Statistical analysis: paired *t*-test (A-B).

**Supplementary Figure 7.**

(A) Heatmap of RNA-seq from T cells co-cultured for 4 days with MV4-11 *TP53* KD or WT (E:T 1:6, 5 ng/ml AMG 330). The heatmap shows hierarchical clustering of significantly upregulated genes in T cells after co-culture with *TP53* KD. *P*_adj_<0.05.

1. Yamada K, Saito M, Matsuoka H, Inagaki N. A real-time method of imaging glucose uptake in single, living mammalian cells. Nature protocols 2007;2:753-762.

2. Schittenhelm MM, Kaiser M, Győrffy B, Kampa-Schittenhelm KM. Evaluation of apoptosis stimulating protein of TP53-1 (ASPP1/PPP1R13B) to predict therapy resistance and overall survival in acute myeloid leukemia (AML). Cell Death & Disease 2024;15:25.

3. Benmebarek, M.R., et al., *A modular and controllable T cell therapy platform for acute myeloid leukemia.* Leukemia, 2021. **35**(8): p. 2243-2257.
