# Supplementary figures and images for: TP53 deficiency in AML induces resistance to T-cell engagers through an immunosuppressive secretome
Source: Leukemia. 2026 Jun 1;40(8):1624–35. doi: 10.1038/s41375-026-02991-6 (PMC13421328; doi:10.1038/s41375-026-02991-6)

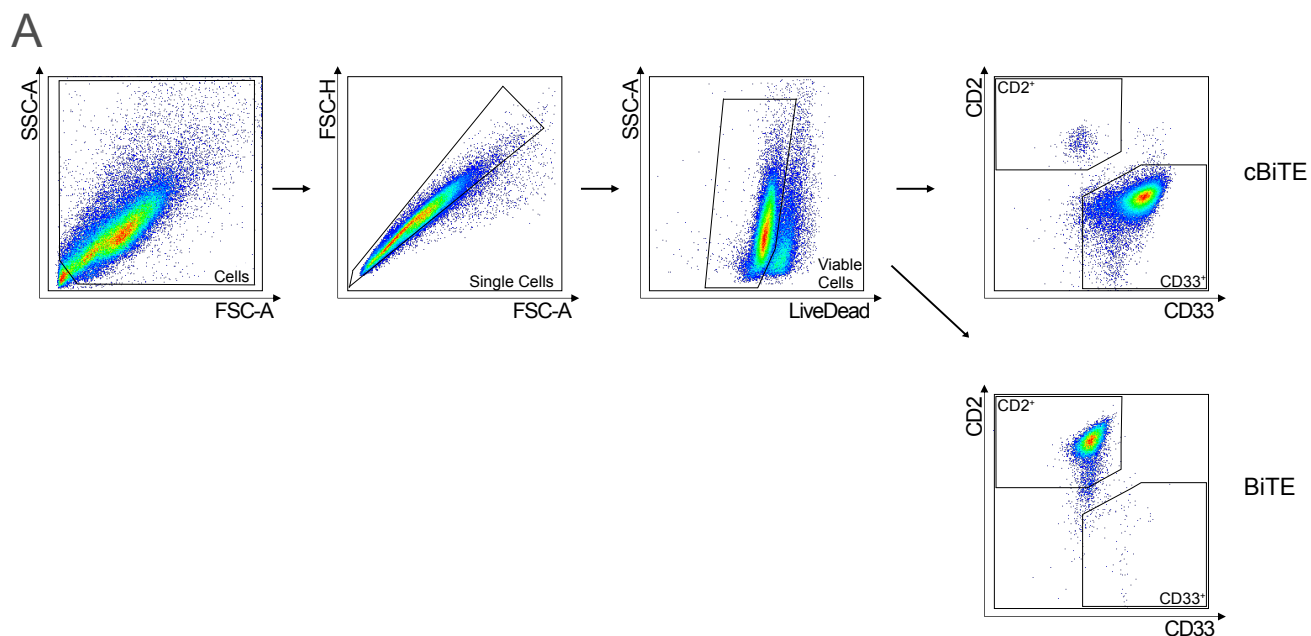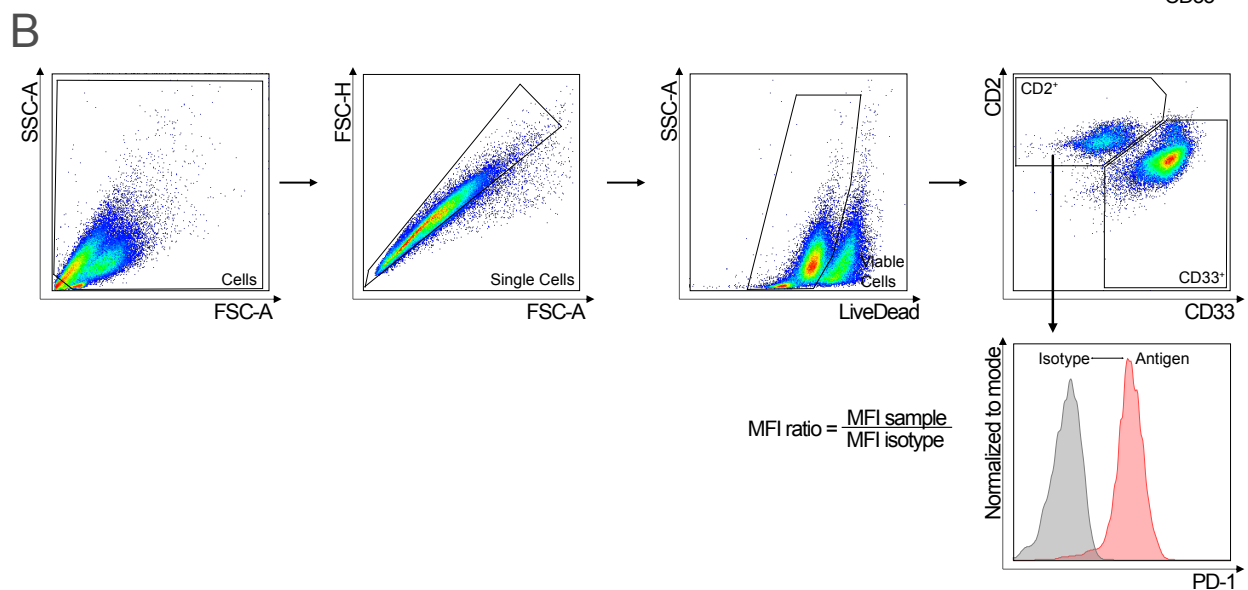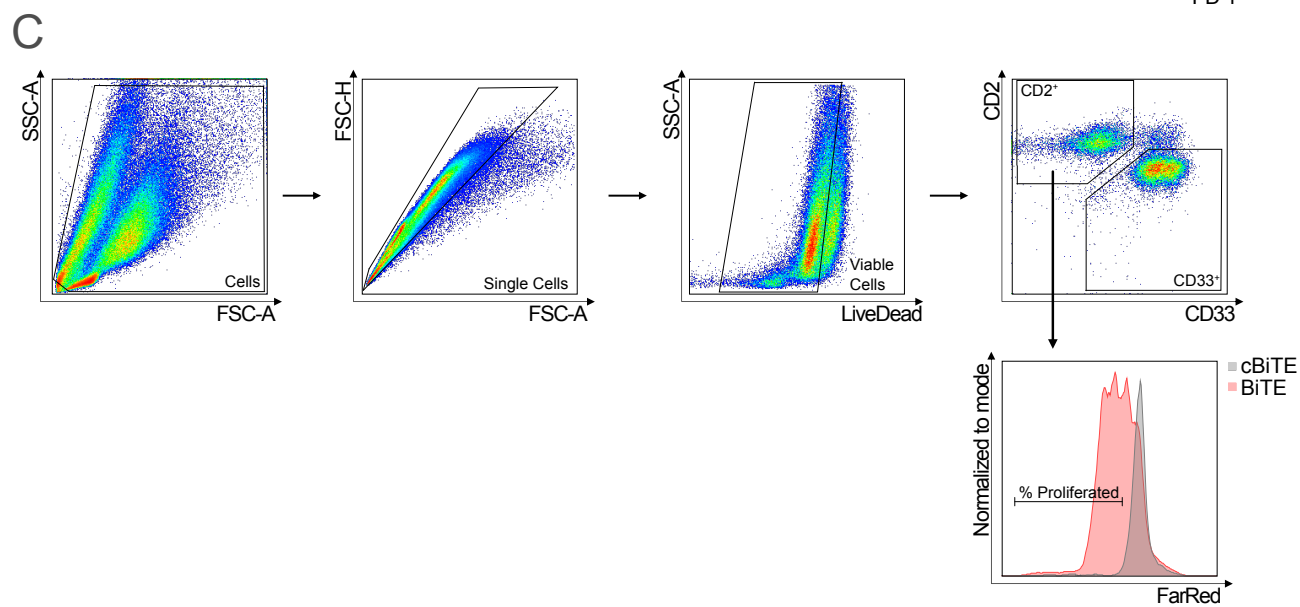

Supplement: Supplementary file 2 — Supplementary Figure 1 [file 41375_2026_2991_MOESM2_ESM.pdf]

■ *TP53* aberration  
 ■ *TP53* KD

A

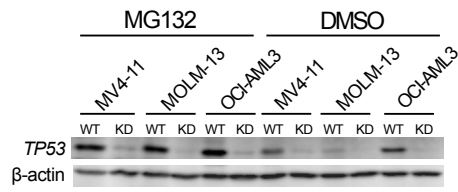

B

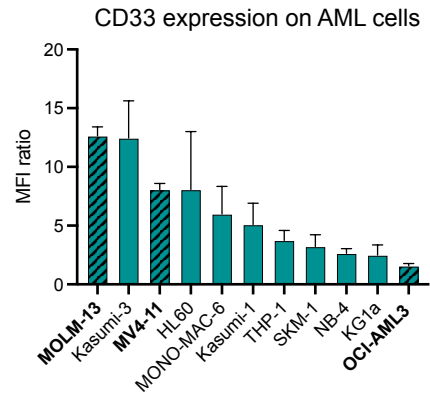

C

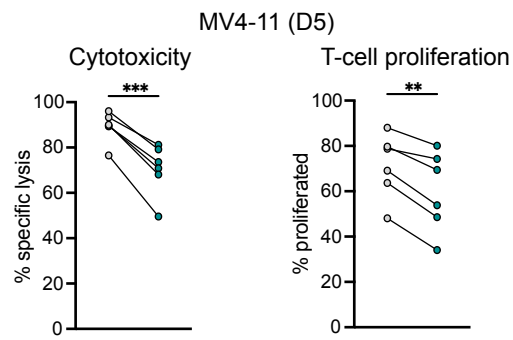

D

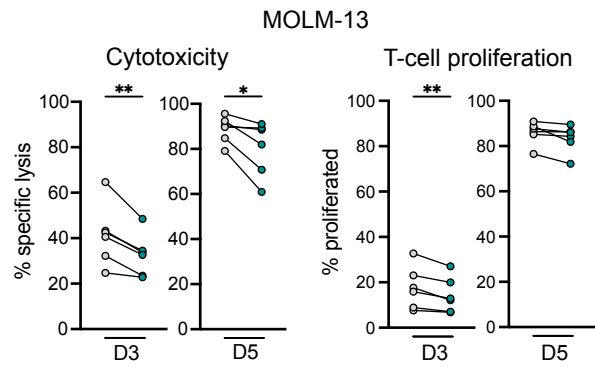

E

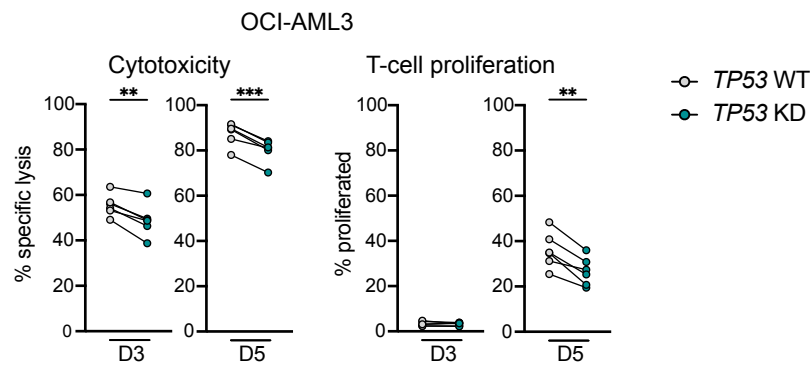

Supplement: Supplementary file 3 — Supplementary Figure 2 [file 41375_2026_2991_MOESM3_ESM.pdf]

TP53 WT  
TP53 KD

A

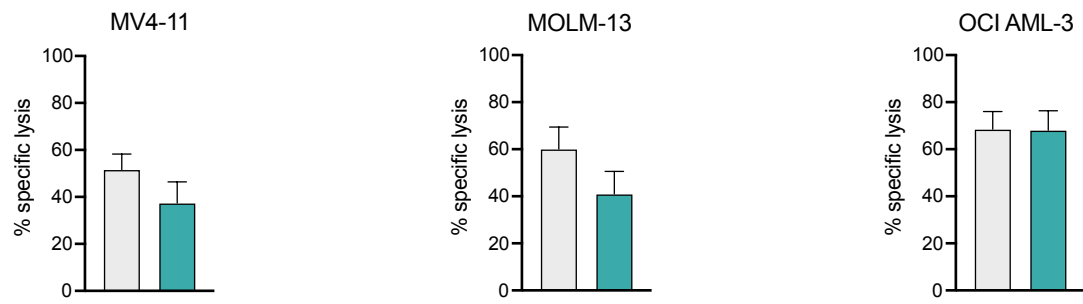

B

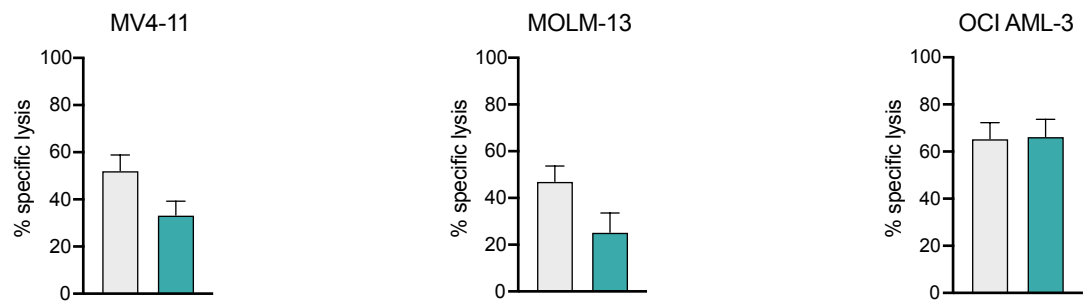

C

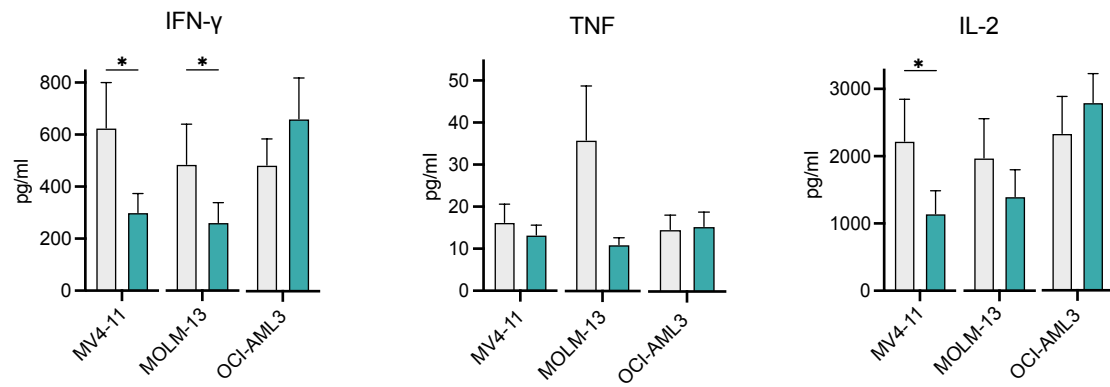

Supplement: Supplementary file 4 — Supplementary Figure 3 [file 41375_2026_2991_MOESM4_ESM.pdf]

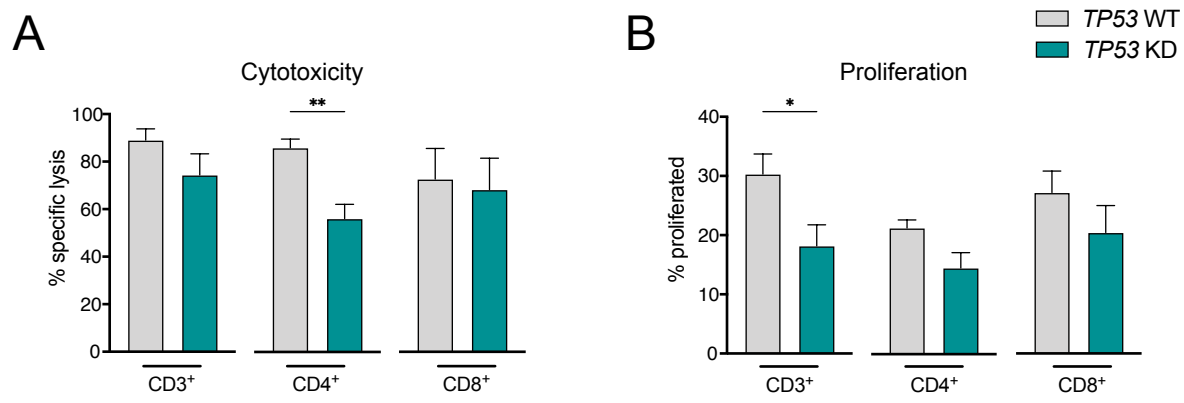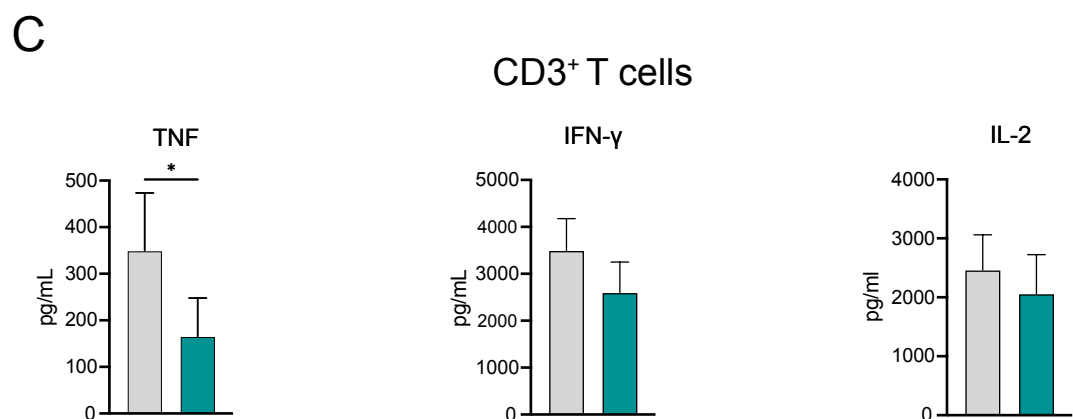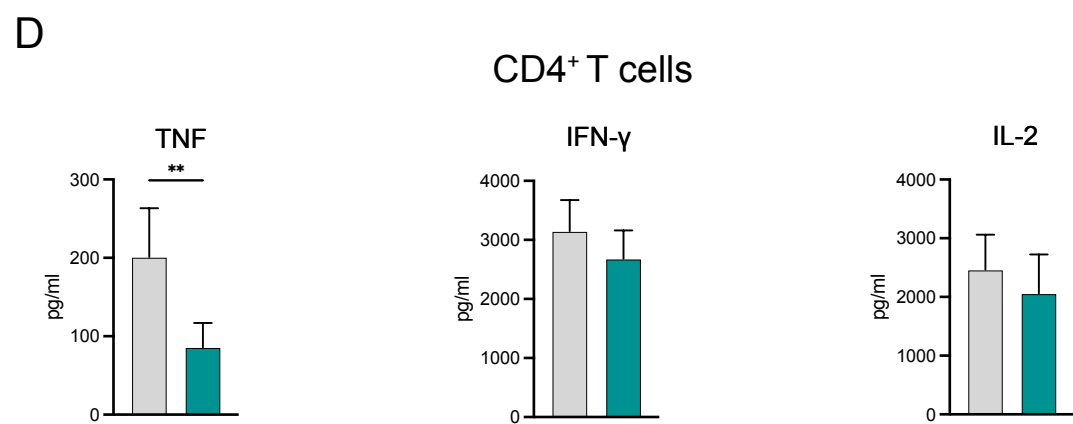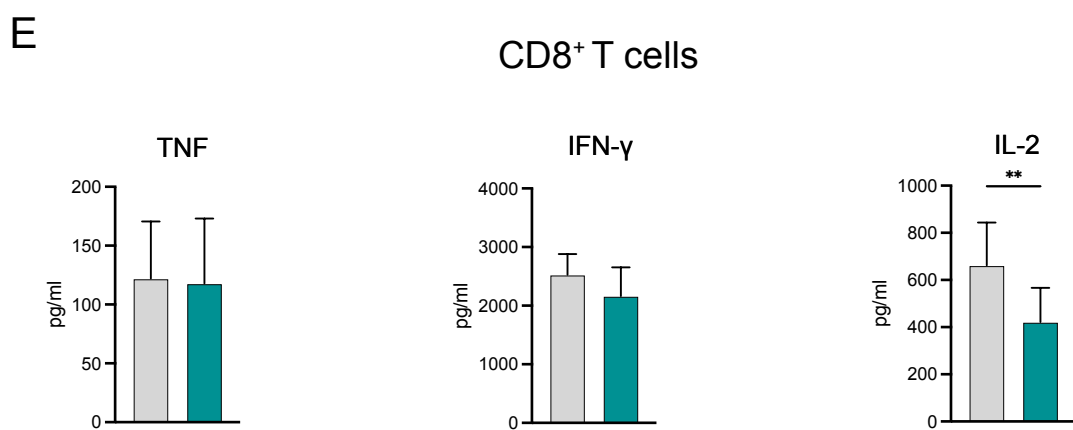

Supplement: Supplementary file 5 — Supplementary Figure 4 [file 41375_2026_2991_MOESM5_ESM.pdf]

A

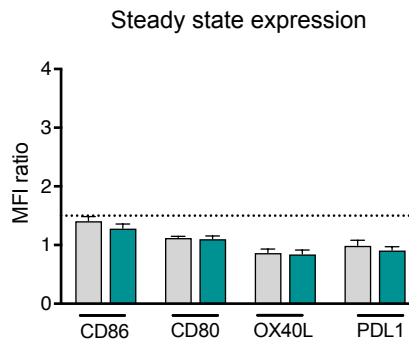

B

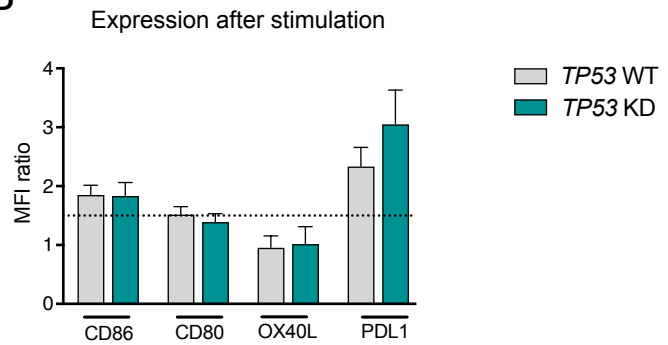

C

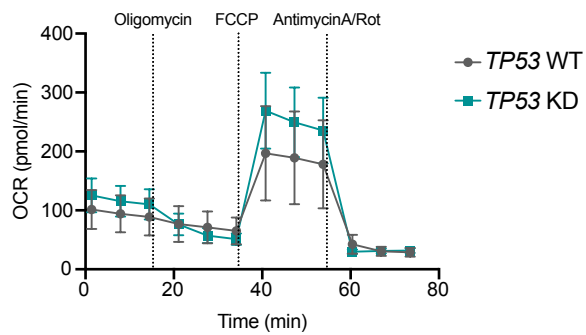

D

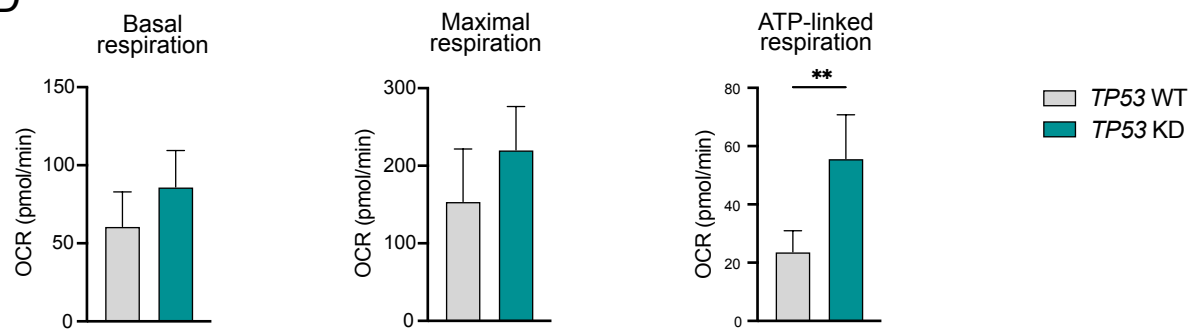

E

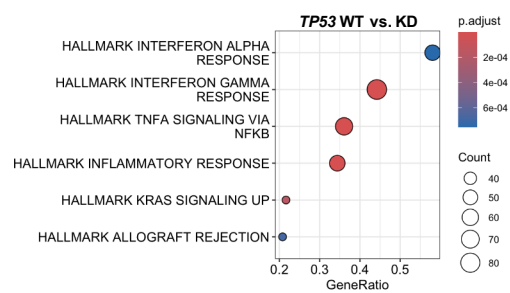

Supplement: Supplementary file 6 — Supplementary Figure 5 [file 41375_2026_2991_MOESM6_ESM.pdf]

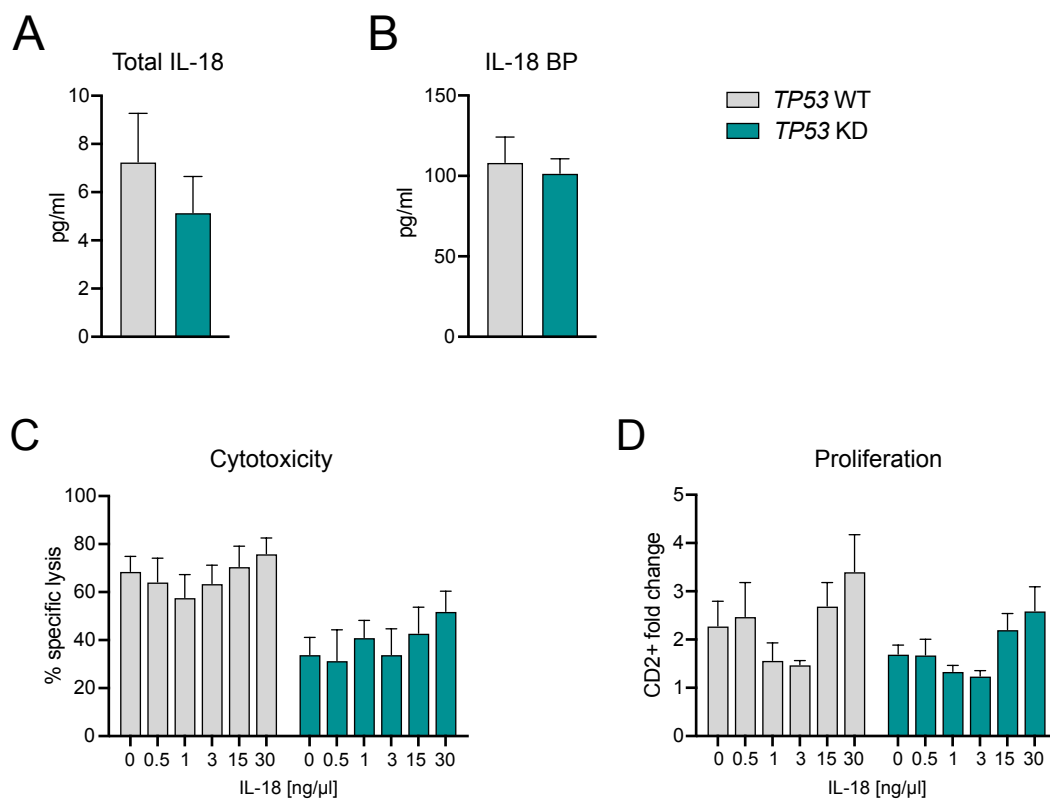

Supplement: Supplementary file 7 — Supplementary Figure 6 [file 41375_2026_2991_MOESM7_ESM.pdf]

A

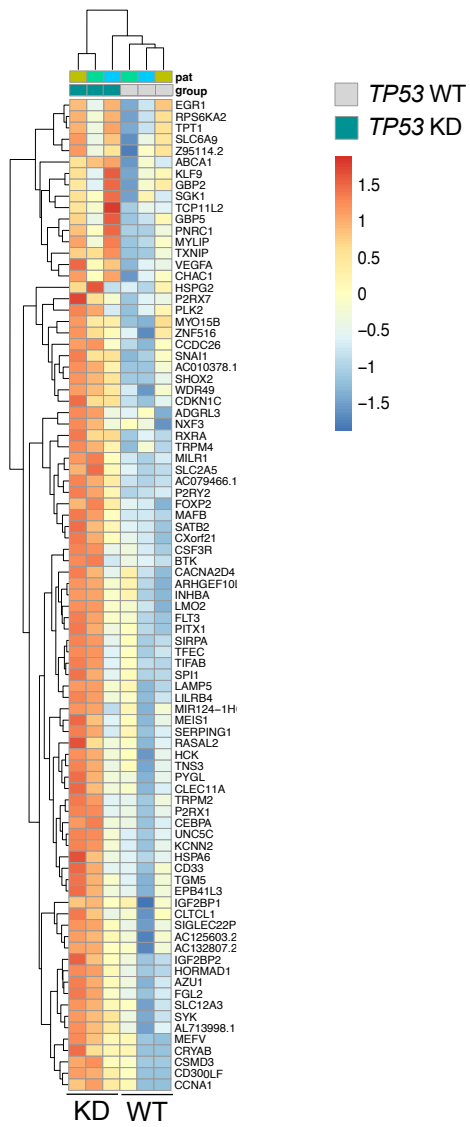

Supplement: Supplementary file 8 — Supplementary Figure 7 [file 41375_2026_2991_MOESM8_ESM.pdf]
